# An efficient hybrid CNN–transformer framework for real-time weapon detection and face recognition

**DOI:** 10.3389/frai.2026.1841848

**Published:** 2026-06-10

**Authors:** P. Shanthi, V. Manjula

**Affiliations:** School of Computer Science and Engineering, Vellore Institute of Technology, Chennai, Tamil Nadu, India

**Keywords:** convolutional neural network, detection transformer, face recognition, vision transformer, weapon detection

## Abstract

The growing demand of smart surveillance systems necessitates the accurate and real-time detection of weapons and face recognition with robustness against occlusion, illumination changes, and complex backgrounds. Existing techniques based on standalone CNN or transformer architectures are less effective in capturing local fine-grained features as well as long-range dependencies. This paper presents ConViDeTR, a hybrid deep learning framework that integrates CNN, Vision Transformer (ViT), and Detection Transformer (DETR) architectures into a unified framework. The key contribution of the proposed framework is the deep feature fusion layer, which integrates local spatial features, global context features, and object query features in one shared feature space. This enables the synchronous execution of weapon detection and face recognition using one efficient framework. The experiments using existing benchmark datasets validate the performance, achieving 98.9% accuracy in weapon detection and 97.34% accuracy in face recognition and outperforming the existing techniques in both tasks. The framework also demonstrates real-time performance with 25–30 FPS and low latency. The performance of the proposed framework sustains its effectiveness, robustness, and scalability in the development of next-generation intelligent surveillance systems.

## 1 Introduction

The intelligent surveillance system developed for modern security are among the most essential technologies globally due to its ability to automatically detect threats and identify individuals in real time. The process involves public safety monitoring and smart cities, and the accurate recognition of weapons reflects the industry’s idea of the connection between digital protection and human safety. Advanced and robust surveillance software systems include applications such as face recognition and access control; however, they also face challenges such as occlusion and illumination variations.

Traditional approaches use numerous architectures in detection, for instance, Convolutional Neural Networks (CNNs) to represent edges, textures, and shapes. These frameworks extract spatial features from local patterns and objects in dynamic scenes. CNN models provide strong capability for extracting local features; however, CNNs are limited in capturing long-range dependencies, whereas transformer-based models effectively capture global contextual interactions.

With the advancement of digital and IP-based CCTV systems, improved resolution video, remote access, and network capabilities are now available. Today, a modern analytic model provides advanced video analytics by detecting, recognizing, and tracking objects for safety monitoring or crime reduction purposes (Tsakanikas and Dagiuklas, 2018; Coudert, 2009; Rai, 2019). An advantage of using deep learning methods in a new camera surveillance system is the ability to analyze and evaluate video stream feeds for suspicious behavior that would warrant notifying the police immediately (Foresti, 1998). Some deep learning models for surveillance can detect guns and knives and provide an immediate notification of potential danger and recognize individuals by cross-referencing their faces with known crime suspects. Deep learning detection models provide immediate identification of potential threats and suspects, supporting proactive crime reduction and enforcement strategies in congested or high-crime areas. Despite recent improvements in surveillance systems, current methods have three main problems: (i) they struggle to effectively combine local and global feature representations, (ii) their accuracy drops with occlusion and complex backgrounds, and (iii) they lack unified frameworks that can perform weapon detection and face recognition at the same time.

In contrast to existing hybrid models, the ConViDeTR framework employs a unified feature fusion method. This approach combines CNN feature maps, Vision Transformer embeddings, and DETR object queries into a single, shared representation. This design allows for simultaneous detection and recognition, which reduces unnecessary computations. The main contributions of the ConViDeTR method are summarized in the following:

- A tightly coupled parallel hybrid architecture integrating CNN, ViT, and DETR into a unified end-to-end framework.
- A unified feature fusion framework that maps local, global, and object-level features into a common embedding space for coherent learning.
- The multi-task unified approach that carries out weapon detection and facial recognition in a single architecture.
- Joint learning of the spatial aspect, global context, and semantic relation of objects, which enhances the cross-level interaction of features.
- The unified pipeline to decrease redundancy and simplify the overall architecture for better performance in real-time deployments.

To tackle these issues, this paper introduces ConViDeTR, a hybrid deep learning framework that combines CNN, Vision Transformer (ViT), and Detection Transformer (DETR) into one architecture. By using the key features of these models, the framework improves both how well features are represented and how efficiently tasks are performed.

The input data of real-world CCTV surveillance can be affected by many different types of degradation such as motion blurring, compression artifacts, and low resolution because of environmental and hardware limitations. As a result, robustness against degraded conditions is an essential requirement for any practical surveillance system. In this regard, the ConViDeTR framework proposed in this paper was developed to address the challenges of CCTV surveillance by taking advantage of the strengths of CNNs for local feature extraction and transformers for global contextual understanding, which allows for performance stabilization even in visually adverse conditions.

## 2 Related works

Arockia Abins et al. (2024) developed a real time weapon detection system using YOLOv5, a deep learning object detection model that performs well detecting firearms and knives in both CCTV and non-CCTV environments. The model has about 87.7 million parameters, and its architecture is comprised of a backbone model based on the CSP (Cross Stage Partial Network), a neck based on PANet (Path Aggregation Network), and a detection head. The system contains weapons detection in real-time with deep learning techniques while achieving the speed of 0.05 s per frame. The system uses a subset of Glenn’s object detection model with CNNs and a PELSF-DCNN classifier that detects weapons while minimizing false positives. A behavior analysis module analyzes the detected objects’ behavior to assess threats, while an alert module alerts local authorities about incidents. The sliding window process and feature selection methods support the system effectively. Nikkath et al. (2022) proposed object detection algorithms of two types: one is based on classification recurrent convolutional neural networks (RCNN), and the other is based on regression. YOLOv5, which is a single-shot detection algorithm, uses bounding box concepts and fully automates surveillance with smart video capturing capabilities using deep learning techniques to remotely monitor strange activities with the exact location and time of the event, along with weapon detection and facial recognition of the criminal.

Tabassum et al. (2023) developed a low-cost and efficient AI-based method for recognizing weapons in surveillance videos in real-time and in different environments. The system is verified and makes use of TensorFlow technology. This application utilizes two distinct dataset types. One dataset contains pre-labeled pictures, whereas the other requires manual label addition. This method is applicable everywhere on the globe. We utilize a range of algorithms, including the Single Shot Multi-Box Detector (SSD), a well-known object detection technique, and Mobile Net, which is a convolutional neural network (CNN) feature. We use different weapon datasets with more than 2000 gun images, which are taken from the COCO dataset.

Kumar et al. (2020) employed facial recognition technology to assist police in real-time criminal identification from CCTV footage or manual images. It automates the process of matching faces captured from CCTV footage or manual images against a pre-existing database of criminal faces. In face detection, it is used as a Haar Feature-Based Cascade classifier. This method uses machine learning to detect faces by training a cascade function on positive (face) and negative (non-face) images, and face recognition, used as Local Binary Pattern Histogram (LBPH), analyzes the local features of an image to compare and identify faces effectively.

Kumar et al. (2023) state that facial recognition in criminal identification helps law enforcement detect and recognize faces from images or videos in real time, providing critical support. The Multi-Task Cascaded Neural Network (MTCNN) is used for face detection, while the Siamese Neural Network identifies faces through one-shot learning. Techniques include preprocessing images, feature extraction, and database matching. The methodology comprises three layers: P-Net (Proposal Network) for initial face detection, R-Net (Refinement Network) to refine results, and O-Net (Output Network) for final face localization and recognition. Nagnath et al. (2021) system locates and isolates the facial region within the pre-processed image using knowledge-based methods to identify key facial boundaries and landmarks. During feature extraction, Principal Component Analysis (PCA) converts the detected face into a mathematical representation that creates eigenfaces, which are the basic components for recognition tasks. The extracted facial features are converted into numerical vectors and compared against the existing criminal database using eigenface templates to calculate similarity scores. The system displays matching criminal records back to the users once similarity scores cross the 70% mark and then grants law enforcement potential suspects.

Ahire et al. (2023) studied various facial detection and machine learning techniques for criminal identification, aiming to improve metropolitan safety through computationally accurate crime predictions and trend analysis. To collaborate on multiple machine learning algorithms, including linear regression, SVM, Naive Bayes, KNN, decision trees, random forests, K-means clustering, and neural networks to detect both criminals and potential crime hotspots. The methodology follows a structured approach incorporating data collection, preprocessing, crime analysis, model development, testing and validation, and system integration for facial recognition-based criminal identification, as in Table 1.

**Table 1:** Analytical literature review of weapon detection and face recognition.

References	Approach	Techniques	Datasets	Findings	Limitations
Elhoseny et al. (2024)	Deep Neural Network (DNN) Conditional Generative Adversarial Network (CGAN) Transfer Learning (VGG19, VGG-16, ResNet-101)	Anomaly detection Techniques Image processing Techniques Facial expression recognition	UCFCrime CelebA FER-2013 YouTube Video	To detect anomalies in the activities of masked individuals. The CGAN architecture successfully generates accurate and realistic facial features for masked individuals, aiding in identity recognition. Achieved 90% for detecting anomalies in masked individuals.	Dataset challenge for masked individuals. Limited by computational resources for real-time video processing. Performance varies with environment
Castillo et al. (2019)	CNN DaCoLT Region Proposal Network (RPN)	Selective search Alarm activation system	Custom Dataset (types of knives and handling scenarios)	The proposed R-FCN model with ResNet101, enhanced by the DaCoLT preprocessing method, achieved a 93% F1 score, reducing brightness-induced performance gaps from 15 to 3%, and enabling real-time detection with an average alarm activation time of 0.41 s.	Manual, time-consuming dataset creation and Struggles with occlusions, distances.
Yadav et al. (2024)	YOLOv7-DarkVision	Brightening algorithm, gamma correction, contrast and brightness adjustment, Gaussian blur, and normalization for low-light enhancement.	Self -recorded YouTube	The YOLOv7-DarkVision effectively detects handguns in low-light conditions, improving visibility and detection accuracy. Integration of brightening algorithms significantly enhances the detection in dark scenarios achieved the accuracy rate of mAP@0.5:95.74%	Performance highly dependent on the brightening algorithm for low-light conditions. Limited real-world testing beyond controlled scenarios.
Yadav P. et al. (2025)	YOLOv7	WeaponVision AI enhances surveillance by effectively detecting firearms in diverse conditions, achieving 91.75% precision and 92.15% mAP.	5 datasets: Pistol Detection YouTube Gun Detection Mock Attack Weapon Detection self-created	Automated alarm system to integrate with surveillance cameras for proactive weapon detection.	The models performance may be affected by the quality of input data and may struggle with occlusions or complex backgrounds.
Dave et al. (2022)	Siamese Neural Network with FaceNet for face embedding.	One-shot learning for face recognition. Haar Cascade Classifier for face detection. Euclidean distance and triplet loss for measurement.	Small dataset: 10 individuals (2 authors and 8 celebrities), with training using 1–3 images per class.	The system effectively identifies criminals with minimal training images using a one-shot learning approach. Achieved testing accuracy of 82.17% with one image per class and 90.01% with three images per class.	Accuracy is lower compared to traditional methods with large datasets. Performance relies on controlled conditions and may not generalize to large-scale or diverse datasets.
Alzu’bi et al. (2025)	MFI3D framework with synthetic face masking GAN-based unmasking 3D face reconstruction	VGG-19 for feature extraction 3D Morphable Models (3DMMs) for facial geometry	CelebAMask-HQ Labeled Faces in the Wild (LFW)	MFI3D achieved an accuracy of 86.60% and a precision of 83.50%, demonstrating strong performance in masked face identification. Integration of 3D reconstruction enhances reliability and robustness against occlusions.	Synthetic data may not fully represent real-world complexity.
Kumar et al. (2024)	CNN, Bi-LSTM, and attention mechanisms	Pre-trained InceptionResNetV3 CNN. Temporal Modeling	UCF11 UCF50 UCF-Crime	Achieved state-of-the-art accuracy: 98.95% on UCF11, 96.94% on UCF50, 62.04% on UCF-Crime	Lower accuracy on the UCF-Crime dataset indicates challenges in highly variable or long surveillance scenarios.
Jain et al. (2020)	Attention SqueezeNet (AttSNet), Bi-LSTM, Adaptive ResNet (A-ResNet), GAN, Hybrid Wild-Hunt (WiH)	Encoder Decoder Forgery Localization Optimization	FaceForensics++	Achieved 96.1% accuracy for localization and 95.4% for detection. Demonstrated superior performance compared to traditional methods across metrics like precision (95.62%) and IoU (91.47%). Reduced computation time to 1.435 s for detection and localization.	Evaluation conducted on a single dataset, limiting generalizability to diverse real-world scenarios. Performance under extreme distortions (e.g., severe noise, compression) not fully explored.

## 3 Proposed methodology

To achieve proposed ConViDeTR framework employs a multi-stage approach as in Figure 1 video surveillance obtained through multiple surveillance cameras was reviewed in stages to achieve processing; preprocessing, annotation for training data, hybrid techniques to extract feature types from images, detection of identified features, and providing an identity verification method will be described thoroughly in this section.

**Figure 1:**
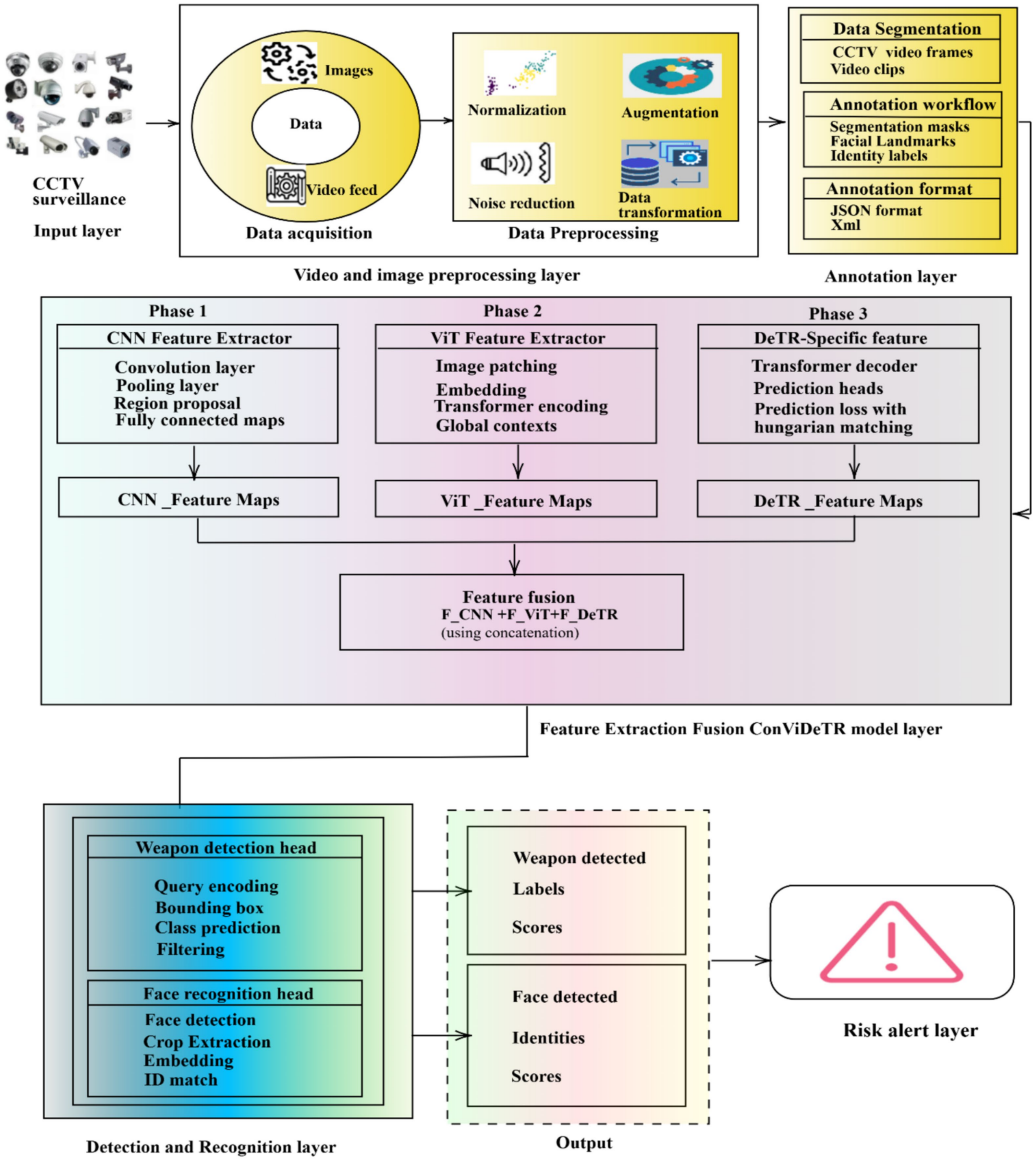
ConViDeTR hybrid detection proposed system.

In addition to increasing robustness against degraded inputs such as motion blurring and compression artifacts, the proposed hybrid CNN + ViT architecture will provide an improved detection and recognition capability by capturing fine details using CNNs, while ViTs will be used to model the sign of correlation among objects and their surroundings.

### 3.1 Input layer

The initial layer of our proposed system is where various visual sensors deployed in the environment capture real-time video footage of incidents. The collected data is then forwarded to the video and image processing layer. High-definition CCTV surveillance cameras are primarily used to capture video and images in real-time.

### 3.2 Video and image processing layer

The surveillance camera’s video stream comprises multiple image frames independently captured during the real-time monitoring process. These frames undergo preprocessing before going to the annotation layer, including resizing, scaling, brightness enhancement, filtering, and sharpening. Preprocessing addresses variations in the size and proportions of input frames to optimize model performance. For uniformity, frames are resized and rescaled, while brightness enhancement techniques improve visibility in low-light conditions, which helps identify key areas within frames. Data augmentation techniques the flipping and various transformations exposed the model to different perspectives about the training sets, enhancing both adaptability and robustness, while normalization reduced sensitivity to possible variations in the pixel intensity along with faster converging during the training process of the pixel intensity. Crime detection, one of which is the ViT model for detecting complex visual patterns. The ViT model was trained on different datasets with images of different sizes and shapes to process the extracted frames from video streams with a high degree of accuracy. It uses self-attention mechanisms and transformer-based architectures that help the ViT model in detecting small features such as weapons, behaviors, crowd monitoring, and face recognition in frames. The model based on labeled data stored in YAML files has been applied to classify and isolate objects, for instance, weapons, with precision.

### 3.3 Annotation layer

Labeling processed data with meaningful information is critical to the guidance of deep learning models. In images and videos, for instance, bounding boxes can be used to outline objects, which could be weapons; assign labels to actions that could be suspicious behavior; and mark points of interest such as facial landmarks, persons, or groups to determine density, hence crowd monitoring or training models to recognize activities. This annotation process organizes the data, which will be ready for supervised training. This step prepares the models to learn patterns and make absolute predictions. The proposed annotation can be divided into three segments: data segmentation, annotation workflow, and annotation format.

#### 3.3.1 Data segmentation

Data segmentation is very essential in handling big data, especially for applications such as weapon detection and face recognition. Data segmentation can be divided into two: feature extraction and specific task segmentation. Feature extraction refers to the breakdown of a video into individual still images or smaller clips. Tools such as OpenCV, FFmpeg, or video processing libraries are typically used, and the process involves breaking a dataset into smaller, more manageable chunks so that easier annotation and analysis can be done. For videos, segmentation entails extracting individual frames or dividing them into short clips, such as one second per interval for 30 frames per second, to focus on certain events. Data splits into sub-datasets meant to be used for the application: weaponry labeling, abnormality detection, tracking density, tracking crowd density, and tracking illegal activities. Datasets split additional crops and tag a face database cropped in all environmental conditions. It enhances training performance from the data model; in other words, it brings a systematic means of bringing ready-task annotated datasets. This may even be handled as a function automatically by importing such Python packages and modules, including Pandas, NumPy, or custom scripts.

#### 3.3.2 Annotation workflow

Data annotation is labeling data to meet detection objectives using specific techniques. For weapon detection, bounding boxes react to “featured” weapons and segmentation to other weapons that are hidden or partially occluded. In suspicious activity analysis, keypoint annotation is employed to label body joints for tracking motion, and activity tagging is used to label the type of activity, “loitering” or “fleeing.” Crowd surveillance is done on the basis of density maps to estimate the number of individuals and heat maps to identify high-risk zones. For detecting facial features, annotation tracks an individual over locations, while behavior labels detect coercive or aggressive interactions, helping with efficient model training.

#### 3.3.3 Annotation format

The goal of annotation formatting is to save annotated data in organized, machine-readable formats for optimal processing. Annotation formatting can be split into three sections. 1. Image data annotation with structured data: Data formats such as JSON, XML, or COCO are employed to structure data with bounding boxes, labels, or segmentation masks. 2. Time-based annotation of video: annotations are maintained in CSV or similar formats along with timestamps to enable time-based monitoring of events. 3. Proper formatting enables machine learning compatibility and effortless parsing of data.

### 3.4 Feature extraction: fusion layer of the ConViDeTR model

The proposed unified multi-task fusion framework differs significantly CNN + ViT hybrid setups or DETR-style detectors. The original contribution of this paper is that a parallel fusion of local (CNN) and global (ViT) features occurs in one representation, which is then input into a DETR-style query decoder that supports both spatial reasoning and identity-aware detection simultaneously. This integrated architecture will provide better contextual comprehension while decreasing redundancy when compared to sequential or loosely coupled architectures.

#### 3.4.1 CNN-based layered extraction of visual patterns

**Figure 2:**
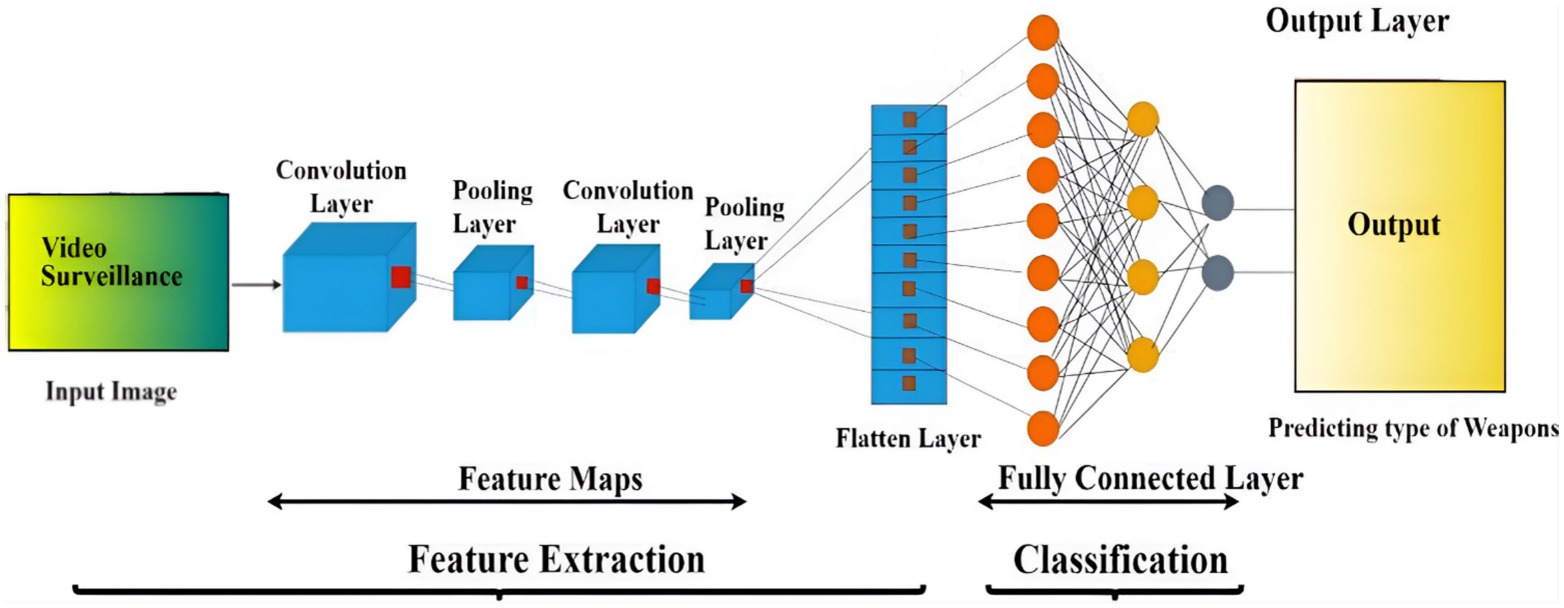
Convolutional neural network.

The first and most vital step in Convolutional Neural Network (CNN)-based weapon detection and face recognition is feature extraction. CNNs can extract meaningful feature maps from raw image pixels through increasingly abstract data representations based on a stack of convolution, normalization, activation, or pooling layers as shown in Figure 2. The first convolution layer consists of learnable filters (kernels) that convolve over the input image, and each filter detects local features such as edges, corners, or textures. For weapon detection, the CNN may capture the edges of a barrel, the texture of a blade, or the shapes of a trigger. Given an input image $I\in R^{C_{\text{in}}\times H\times W}$ , where $C_{\text{in}}$ =3 (the RGB channels), the output of the convolution operation can be represented as in Equation 1:


$$F_{i,j}^{\left(k\right)}=\sum_{m=1}^{M}\sum_{n=1}^{N}\sum_{c=1}^{C_{\text{in}}}K_{m,n,c}^{\left(k\right)}.I_{i+m,j+n,c}+b^{\left(k\right)}$$ (1)

$K^{\left(k\right)}:$ learnable kernel/filter of size $M\times N\times C_{\text{in}}$ , $b^{\left(k\right)}$ : bias term for the k^th^ feature map, $F^{\left(k\right)}$ : feature map after convolution.

The CNN processes different hierarchical representations—relatively low-level features (edges) are salient in early layers, while deeper layers of the neural net provide high-level semantic features (gun silhouette, full face) representations. Pooling allows for some improvement in the translation invariance of the CNN to aspect ratios (e.g., a weapon can still be detected even if it is slightly shifted) or the aspect ratios of faces, as pooling preserves whatever identity that may be signified despite small pose or alignment variations in Equation 2.


$$P_{i,j}^{\left(k\right)}=\max_{(m,n)\in \Omega }F_{i+m,j+n}^{\left(k\right)}$$ (2)

$P_{i,j}^{\left(k\right)}:$ the pooled feature map value at position (i,j) for the kth feature map, $F_{i+m,j+n}^{\left(k\right)}$ : the input feature map value from the convolution layer at location (i + m, j + n), Ω : The value of the pooled feature map at position (i,j) corresponds to the kth feature map, max: the maximum operator, which selects the largest activation within the pooling window. The feature maps from the final convolution and pooling block are processed and then flattened into a 1D vector in Equation 3. This vector contains a representation of features that are compressed but highly informative. For weapons, the vector contains attribute patterns specific to weapons. For faces, the representation encodes distinctive characteristics related to identities.


$$X=\text{Flatten}(p)\in R^{1\times D}$$ (3)

The 1D vector of flattened features is fed through multiple dense layers, which can be described as each neuron in the dense layer connecting to all of the activations from the previous layers. The dense layers also take the feature activation and merge the local features into a global association in Equation 4. For weapons, differentiate between “gun,” “knife,” or “no weapon.” For faces → embeddings from dense layers compare a known face template with the embeddings for recognition purposes. The final FC layer uses the Softmax activation function as in Equation 5:


Z=W.X+b (4)

$$P(y=i|X)=\frac{e^{Z_{i}}}{\sum_{j=1}^{K}e^{Z_{j}}}$$ (5)

P(y=i∣X) : the probability that the input *X* belongs to class *I,* $Z_{i}$ : The raw score also called logit produced by the model for class *I,* K: The total number of possible classes, e.g., 3 weapon types, or N known identities in face recognition. The output layer provides a confidence score for decision-making (triggering alerts in real-time surveillance). Shallow layers → detect edges, corners, and surface textures for both metallic surfaces of weapons and the facial boundary. Mid-level layers → detect shapes, parts, and regions like gun barrels, knife blades, or face parts ears, nose, eyes. Deeper layers → search for patterns at the semantic level the silhouette of weapons or facial features that were unique. The CNN extracts local and mid-level features. The flattened and embedded feature representations (X) are then processed to enhance their capabilities as input tokens into: Vision Transformer (ViT): enhances the modeling to provide global context modeling (long-range dependencies, inadvertent occlusions). DETR operates to perform set-based inference for detection with classification and bounding box regression.

#### 3.4.2 Hierarchy of vision transformer for non-spatial feature extraction

**Figure 3:**
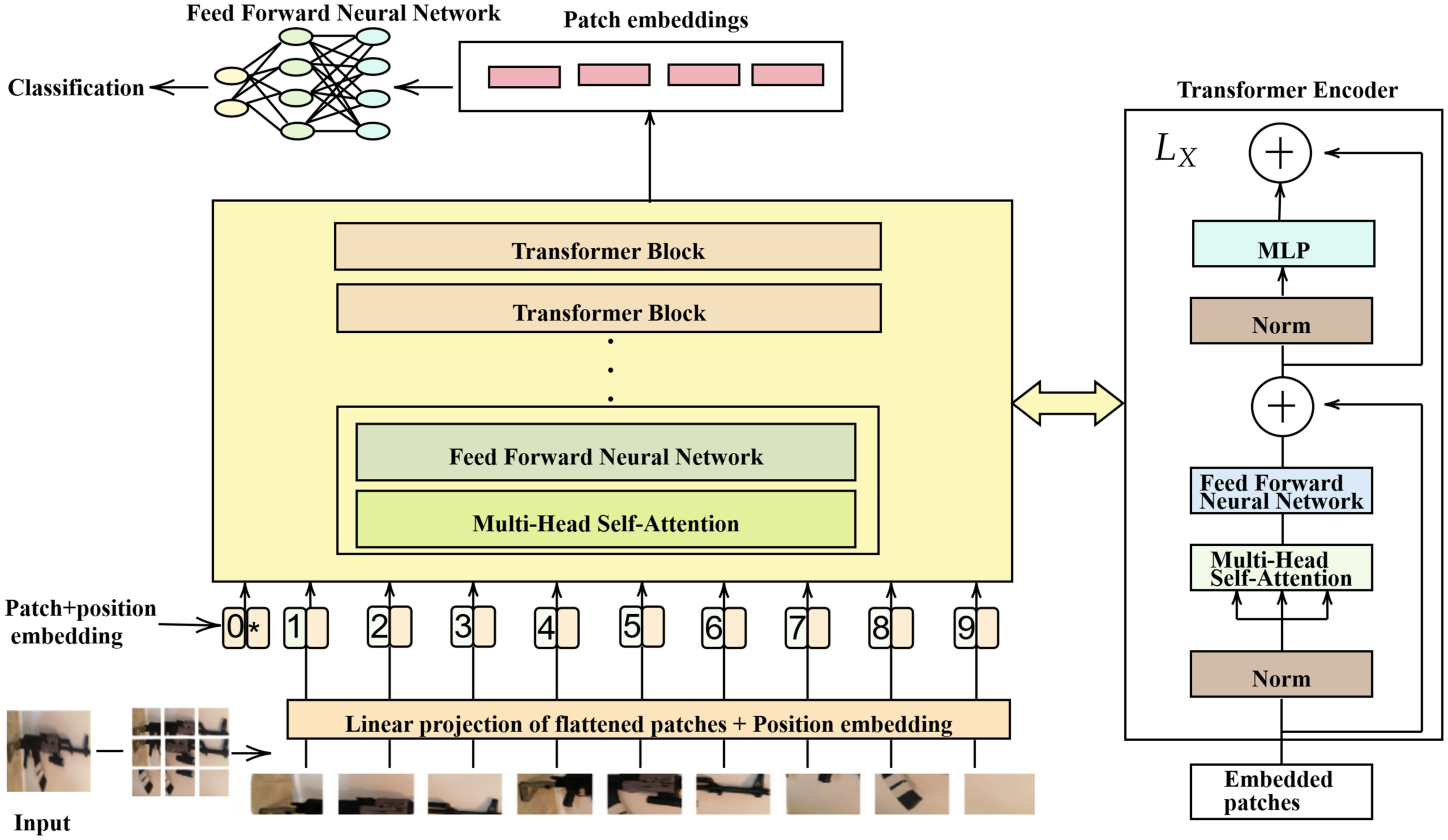
Vision transformer architecture.

The vision transformer breaks up an input image into patches of fixed sizes, adds positional information to the patches formed, and extracts meaningful global features through transformer encoder blocks containing self-attention and MLP layers for classification or detection tasks. This provides an introductory summary of how Vision Transformers migrate the transformer mechanism from NLP to computer vision and highlights the adaptability to model how the transformer uses the global relationships across the image patches for several tasks, as illustrated here in Figure 3.

##### 3.4.2.1 Stage 1: linear projection and position embedding

The raw input image is passed in, and then the patch extraction of the image is split into smaller square-shaped fixed-size patches of 16*16 pixels just for this example. That patch is flattened into a single-dimension vector. For example, if the input image size is H×W (height by width) and the patch is


$$N=\frac{H\times W}{P^{2}}$$ (6)

Where, N —Total number of patches, H —Height of the input image, W —Width of the input image, P —Size of each square patch (P × P). According to Equation 6, this is the number of patches generated when an image of dimension H×W is cut into non-overlapping patches of size P×P . Each patch is then flattened, and a projection layer is applied to yield a patch embedding as in Equation 7.


$$z_{i}^{0}=W.x_{i}+b$$ (7)

Where, $z_{i}^{0}$ : The embedding vector for patch iii, with dimensionality D, $x_{i}$ : The flattened patch i, with dimensionality, W : Learnable weight matrix of shape, b : Bias term. A position embedding vector $p_{i}\;$ of the same size D is assigned to each patch embedding $z_{i}^{0}$ . The matching patch embedding is supplemented with the position embedding as in Equations 8, 9.


$$z_{i}^{0}=z_{i}^{0}+p_{i}$$ (8)

$$z^{0}=[z_{1}^{0}+p_{1},,,z_{2}^{0}+p_{2},,,\ldots ,,,z_{N}^{0}+p_{N}]$$ (9)

The sequence of fixed-dimensional vectors $z^{0}\in R^{N\times D}$ in Equation 8 is then processed by the transformer encoder.

##### 3.4.2.2 Stage 2: transformer encoder

The Multi-Head Self-Attention (MHSA) function is a fundamental feature of the transformer encoder, as it enables the model to capture relationships and dependencies in the input tokens (image patches). Achieving this by assessing all tokens in relation to one another with the assignment of attention weights enables the model to capture the relevant patches as the sequence is processed. Each token is linearly projected into a query (*Q*), key (*K*), and value (*V*) vectors using learnable weight matrices $W_{Q},W_{K,}W_{V,}$ which are optimized during training. The attention output weights combined using an output projection matrix $W_{O}$ in Equation 10. The Feed-Forward Neural Network (FFNN) is a simple two-layer fully connected network applied independently to each token after the self-attention layer. Its goal is to augment feature representation by applying non-linear transformations. The two-layer network in Equation 11 processes each token through and extends model expressiveness through learning of compound features and adds to MHSA by refining the token representations post-capture of dependencies.


$$\text{Attention}(Q,,K,,V)=\text{softmax}(\frac{QK^{T}}{\sqrt{d_{k}}})V$$ (10)

$$\text{FFNN}(x)=\sigma (W_{2}.\text{ReLU}(W_{1}.x+b_{1})+b_{2})$$ (11)

Where $d_{k}$ is the dimension of the keys, $W_{1}$ and $W_{2}$ : Learnable weight matrices, $b_{1}\;\text{and}\;b_{2}$ : Bias terms, σ : Non-linear activation (e.g., GELU or ReLU). The layer normalization (norm) of the input improves convergence during training by reducing the effects of exploding or vanishing gradients. It keeps the input scale consistent, which is essential for the transformer, as even small perturbations may be amplified as they move through layers as in Equation 12.


$$\text{Norm}(x)=\frac{x-\mu }{\sqrt{\sigma^{2}}+\sqrt{\varepsilon }}.\gamma +\beta$$ (12)

$$\mathrm{MLP}(z)=(W_{2}.\text{ReLU}(W_{1}.z+b_{1})+b_{2})$$ (13)

μ : Mean of the input features, $\sigma^{2}$ : Variance of the input features, ε : Small constant added for numerical stability, γ,β : Learnable scaling and shifting parameters. as shown in Equation 13. $W_{1}$ and $W_{2}$ : Weight matrices for the two dense layers, $b_{1}$ and $b_{2}$ : Bias vectors, ReLU : Activation function (alternatively, GELU is used for smoother activation).

##### 3.4.2.3 Stage 3: output of global features

The MLP enhances the representation by learning non-linear combinations of the features extracted by the self-attention mechanism, and it allows projection of the feature vectors to desired dimensions. After passing through the transformer encoder, a [CLS] token embedding (predefined for classification) is passed to a feed-forward neural network for classification and finally prediction probabilities.

#### 3.4.3 Hierarchy of detection transformer (DeTR)

**Figure 4:**
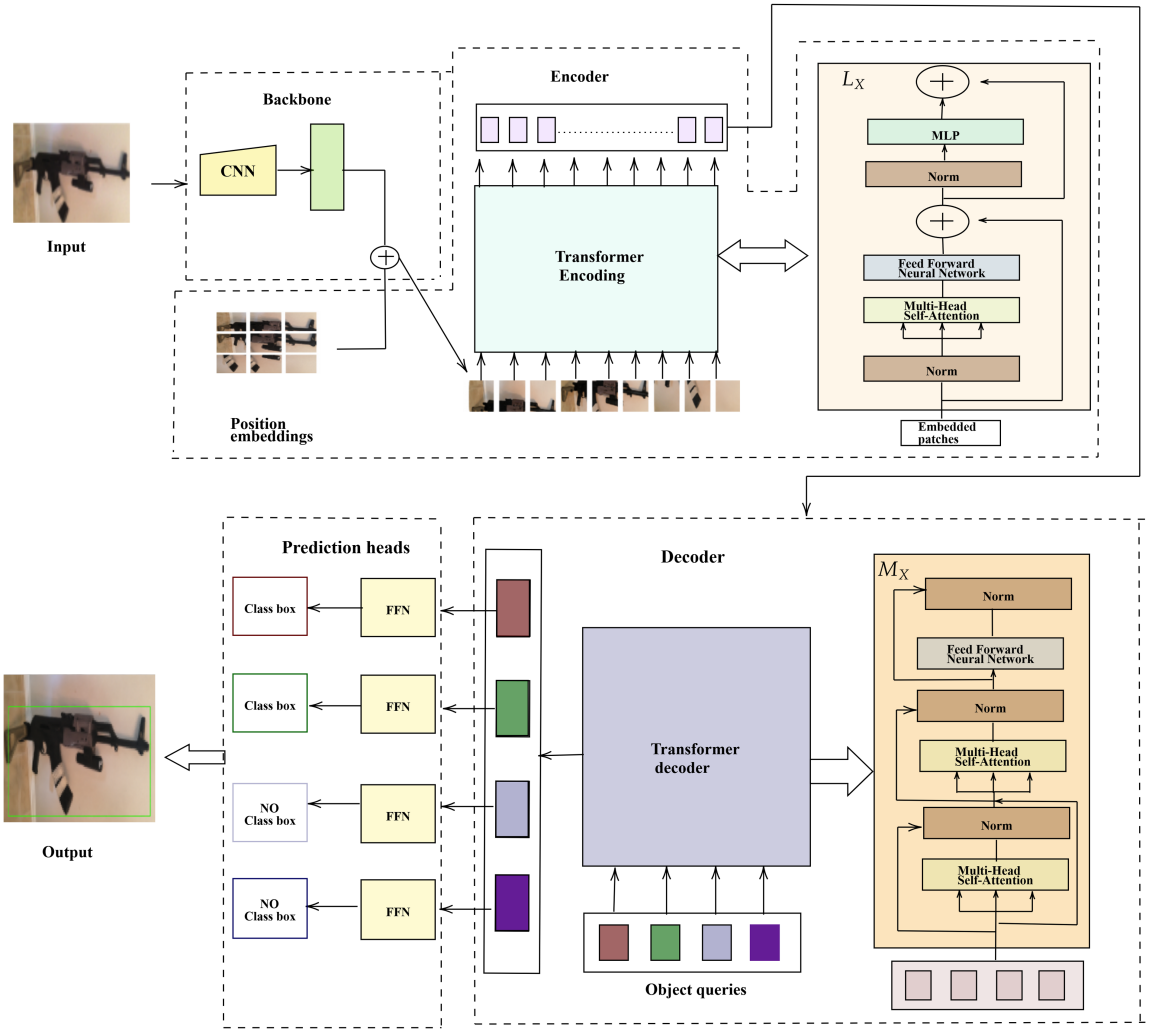
Detection process using the detection transformer.

Object detection seeks to locate and identify objects within an image. RPNs and anchor boxes are the foundation of convolutional models, while transformer-based models dispense with these fixed heuristics by learning object position end-to-end. The Detection Transformer (DETR) is a pure attention-based method that formulates object detection as a set prediction problem, as shown in Figure 4.

The input is an image that goes through a Convolutional Neural Network (CNN) in Equation 14 Backbone, such as the primary network ResNet-50, empowers feature extraction performance and efficiency of computing. Its residual learning structure is very effective for training deep representation while avoiding the problem of vanishing gradients. In terms of computation, the ResNet-50 is less complex than the deeper versions like ResNet-101; therefore, it is better suited for real-time surveillance. Additionally, ResNet-50 has been shown to work well within object detection frameworks and will integrate easily into the transformer-based DETR module used in this study. Position embeddings Equation 15 are added to retain spatial information (since Transformers do not have an inherent sense of position).


F=CNN(I) (14)

$$\hat{F}=F+P$$ (15)

Where, I is the input image (H × W × 3), F is the extracted feature map (H′ × W′ × C), C is the number of channels in the feature map, P is a learned position encoding matrix. The extracted features are flattened into a sequence of vectors and fed into a Transformer Encoder. The encoder consists of Multi-Head Self-Attention (MHSA) layers to model global relationships between image regions as in Equation 16. Feed-forward networks (FFN) tokens are passed through a two-layer feed-forward network for further feature transformation, as in Equation 17.


$$\text{Attention}(Q,K,V)=\text{softmax}(\frac{QK^{T}}{\sqrt{d_{k}}})V$$ (16)

$$Y=\sigma (W_{2}\text{ReLU}(W_{1}X)+b_{1})+b_{2}$$ (17)

Where: Q = $\;XW_{Q}(\text{query}),K=XW_{K}(\mathrm{Key}),K=XW_{V}(\text{value})$ , $d_{k}$ is the query/key dimension. $W_{1}$ , $W_{2}\;$ are learnable weights.

Layer Normalization (Norm) and Residual Connections to balance training. The decoder receives object queries (learnable embeddings) that interact with encoded features. It consists of multiple attention layers: Self-Attention Layers (to capture relationships between queries). Cross-attention layers focus on relevant regions in the encoded image representation as in Equation 18. The output from the decoder is passed to prediction heads, as in Equation 19 these heads classify objects and predict bounding boxes.


$$\text{Cross Attention}(Q_{o},,K,,V)=\text{softmax}(\frac{Q_{o}K^{T}}{\sqrt{d_{k}}})V$$ (18)

$$\hat{b}=\sigma (W_{b}Q_{o}+b_{b})$$ (19)

Where $Q_{o}\;$ typically comes from the decoder, while K (keys) and V (values) come from the encoder, where $W_{b}$ is a learnable weight matrix. Some queries may not correspond to any object (indicating no detection). The final output consists of detected objects along with their bounding boxes. Objects are matched with predicted boxes using the Hungarian algorithm in Equation 20, and DETR uses generalized IoU (GIoU) Loss.


$$L_{\text{match}}=\sum_{i}(L_{\text{class}}(y_{i},\hat{y_{i}})+\lambda L_{\mathrm{box}}(b_{i},\hat{b_{i}}))$$ (20)

Where, $L_{\text{match}}$ :is the total matching cost, $L_{\text{class}}(y_{i},\hat{y_{i}})$ measures the classification loss between the predicted class $y_{i}$ and the ground truth class $y_{i}$ , $L_{\mathrm{box}}(b_{i},\hat{b_{i}})$ quantifies the difference between predicted bounding boxes $\hat{b_{i}}$ and ground truth boxes $b_{i}$ (using a combination of IoU loss and L1 loss), λ is a weighting factor that balances classification and localization loss. The Hungarian algorithm provides a method to find the optimal assignment of predictions to ground-truth objects that minimizes $L_{\text{match}}$ , allowing each detection to match a unique real-world object as in Equation 21.


$$L_{\text{GIoU}}=1-\frac{|A\cap B|}{|A\cup B|}$$ (21)

Where A and B are the predicted and ground-truth boxes. This end-to-end detection model can model global feature interactions, improve spatial reasoning, and achieve competitive accuracy on datasets like COCO, outperforming object detection systems based on traditional hand-engineered CNN representations that use region proposals.

### 3.5 Hybrid CNN-ViT-DeTR framework: weapon detection and face recognition layer

The proposed framework employs a Convolutional Neural Network (CNN), a Vision Transformer (ViT), and a Detection Transformer (DeTR) in a single pipeline to exploit the complementary aspects of each architecture. CNNs are powerful local spatial feature extractors, e.g., edges and texture, but they are inherently limited by their localized receptive fields, which constrain CNNs’ ability to capture long-range dependencies. In contrast, ViTs have displayed impressive abilities to capture global contextual relationships by modeling whole images as a sequence of image patches that can be processed by self-attention. Patch-wise modeling may suppress fine spatial detail, and ViTs typically require large data scales and compute resources to deliver optimal performance. The DeTR module complements retrieval by enabling object-centric reasoning via a transformer decoder with learnable queries, enabling direct classifications and bounding box regression using no handcrafted anchors or region proposals.

To reconcile these trade-offs, we propose a three-dimensional feature extraction mechanism in which the CNNs provide precise local features; the ViTs construct long-range structures to capture contextual dependencies; and the DeTR generates object-level predictions, where the positional and semantic information are aligned. When integrated together, these complementary feature spaces yield a compact yet highly informative representation that can capture both detection and recognition use cases in challenging surveillance settings.

#### 3.5.1 ConViDeTR fusion model

Each of the outputs from the CNN, ViT, and DeTR branches captures different but complementary aspects of visual representation. To bring them into a common space, we project all the feature maps into a common dimensionality via a linear mapping as in Equation 22:


$$\begin{array}{l} F_{\mathrm{CNN}}^{\prime }=W_{c}F_{\mathrm{CNN}}+b_{c},F_{\mathrm{ViT}}^{\prime }=W_{v}F_{\mathrm{ViT}}+\\ b_{v},F_{\text{DeTR}}^{\prime }=W_{d}F_{\text{DeTR}}+(b_{d}) \end{array}$$ (22)

The features are then normalized and dimensionally matched and concatenated together based on the fused representation Equation 23:


$$F_{\text{fusion}}=[F_{\mathrm{CNN}}^{\prime }\|F_{\mathrm{ViT}}^{\prime }\|F_{\text{DeTR}}^{\prime }]$$ (23)

where there || is an operation representing concatenation over the feature dimension. To ensure stable training, we next utilize a layer normalization such that in equation 24.


$$F_{\text{fusion}}=\text{LayerNorm}(F_{\mathrm{fu}s\mathrm{ion}})$$ (24)

Thus, the fused embedding retains local appearance cues (from CNN), global contextual dependencies (from ViT), and object-centric semantics (from DeTR). Although we could have taken a more elaborate path like weighting via attention, instead we found from empirical evaluation that simple concatenation gave the best balance between accuracy and compute efficiency with respect to general applications in real-time surveillance systems Equation 25.


$$F_{\text{fusion}}=\sum_{i\in \{\mathrm{CNN},\mathrm{ViT},\text{DeTR}\}}\alpha_{i}F_{i}^{\prime },\alpha_{i}=\frac{e^{w_{i}}}{\sum_{j}e^{w_{j}}}$$ (25)

#### 3.5.2 Weapon detection head

The weapon detection head uses the fused features $F_{\text{fusion}}$ as its input and uses the DeTR-style query-based prediction process.

##### 3.5.2.1 Query encoding

To introduce a set of learnable queries ${\left\{q_{i}\right\}}_{i=1}^{Q},q_{i}\in R^{d}\;$ which means that we have some number of queries, each of which is associated with a possible object in the scene. Each of the queries evaluates the fused feature representation $F_{\text{fusion}}$ through a cross-attention Equation 26:


$$h_{i}=\text{Attention}(q_{i},F_{\text{fusion}})$$ (26)

Where, $q_{i}$ = the query vector that queries the fused features, $F_{\text{fusion}}$ = the global feature representation that contains CNN (local), ViT (global), and DeTR (object-level) cues, $h_{i}$ = the refined embedding for the i*^th^* candidate, which captures both object-level semantics and, more generally, the spatial context of the candidate.

##### 3.5.2.2 Class prediction

The refined embedding hi, we convert it to class logits by mapping it into the class space through a linear projection and then applying a Softmax activation Equation 27:


$$p_{i}=\text{softmax}(W_{c}h_{i}+b_{c}),p_{i}\in R^{K}$$ (27)

Where: $W_{c}\;\text{and}\;b_{c}$ ​ are learnable parameters of the classification head, K is the number of classes ({gun, knife, no-weapon}), $p_{i}$ provides the probability distribution over classes.

##### 3.5.2.3 Bounding box regression

In addition to the class predictions, another linear projection is applied to each hi to predict the normalized spatial coordinates of the bounding box Equation 28:


$$b_{i}=\sigma (W_{b}h_{i}+b_{b}),b_{i}=(x,y,w,h)\in {\left[0,1\right]}^{4}$$ (28)

Where: $W_{b}$ , $\;b_{b}$ are learnable regression parameters, x,y denote the normalized center coordinates of the bounding box, w,h denote the normalized width and height, σ (sigmoid function) ensures that all outputs lie within [0,1] making them image-size independent.

##### 3.5.2.4 Filtering

The multiple queries produce candidate detections, a filtering step is applied. Only those predictions with class confidence above a threshold *τ* are retained Equation 29:


$$D_{\text{weapon}}=\{(b_{i},c_{i},\max(p_{i}))|\max(p_{i})>\tau \}$$ (29)

where: $c_{i}=$ arg $\max(p_{i})$ is the predicted class label for candidate *I,* $\max(p_{i})$ is the confidence score for that prediction, τ is a predefined threshold (e.g., 0.5).

#### 3.5.3 Face recognition head

The face recognition head uses the fused features to detect, embed, and identify faces of humans in challenging conditions of surveillance, consisting of four main parts: face detection, cropping & alignment, embedding extraction, and identity matching.

##### 3.5.3.1 Face detection

Candidates face bounding boxes are predicted as Equation 30:


$$B_{\text{face}}={\left\{b_{j}\right\}}_{j=1}^{M},b_{j}=(x,y,w,h)$$ (30)

Where, M = number of detected face candidates, x,y = normalized center coordinates of the bounding box., w,h = width and height of the detected region.

##### 3.5.3.2 Cropping and alignment

After obtaining a bounding box, the applicable region can be cropped in Equation 31:


$$I_{j}=\text{Crop}(I,b_{j})$$ (31)

where *I* is the original input frame and $I_{j}$ is the cropped face region. To increase recognition robustness, alignment can be applied (e.g., by moving eye locations) such that scale and orientation are consistent across multiple faces.

##### 3.5.3.3 Embedding extraction

All cropped faces $I_{j}\;$ will then be passed through a hybrid CNN-ViT encoder to produce a compact, discriminative vector that encodes the face’s identity as in Equation 32


$$e_{j}=f_{\theta }(I_{j})\in R^{d}$$ (32)

Where, $f_{\theta }$ = the hybrid CNN–ViT encoder, $e_{j}$ = the d-dimensional embedding vector that encodes the face identity.

##### 3.5.3.4 Identity matching

Each extracted embedding is compared with a stored database of registered faces $\{e_{k}^{\mathrm{DB}}\}.$ The comparison is made by using cosine similarity in Equation 33:


$$S(e_{j},e_{k}^{\mathrm{DB}})=\frac{e_{j}.e_{k}^{\mathrm{DB}}}{||e_{j}||\;|\|e_{k}^{\mathrm{DB}}\||}$$ (33)

where: $e_{j}$ = embedding of the detected face, $e_{k}^{\mathrm{DB}}$ = stored embedding for the *k^th^* registered identity, S (·) = similarity score between [−1,1]. The predicted identity is then assigned as the identity class of maximum similarity in Equation 34.


$$y_{j}=\arg\max_{k}S(e_{j},e_{k}^{\mathrm{DB}})$$ (34)

##### 3.5.3.5 ArcFace margin-based classification

ArcFace increases discriminability by adding an angular margin penalty to the classification loss. This has the effect of forcing the embeddings of the same identity to be more compact and the other (different) identities to be more separable as Equation 35. The ArcFace loss is formulated as follows:


$$L_{\text{ArcFace}}=-\frac{1}{N}\sum_{i=1}^{N}\log\frac{e^{s.(\cos(\theta_{\mathrm{yi}}+m))}}{e^{s.(\cos(\theta_{\mathrm{yi}}+m))}+\sum_{j\neq \mathrm{yi}}e^{\text{scos}\theta_{j}}}$$ (35)

where:

S = scaling factor controlling the magnitude of logits.

m = angular margin that enforces stronger inter-class separability.

$\theta_{\mathrm{yi}}\;$ = angle between the feature embedding and the ground-truth class center.

### 3.6 Risk layer

An alert system typically comprises several levels, which reflect differing levels of urgency and severity of detected risks. For example, in the context of security, the lowest alert level may indicate a minor issue, while the highest alert level indicates a critical incident. In a crime-detection, surveillance-based entity, alerts are organized into five categories and color distinctions: critical—danger, high—severe threat, medium—warning, low—caution, and informational—monitoring. Each alert represents the level of severity related to the detected risk. CRITICAL alerts would be for an active shooter, abduction, etc., that demand immediate action by law enforcement. HIGH alerts would indicate a level of heightened risk that is probable but not imminent, such as concealed armed weapons or aggressive behavior, and would need immediate verification and surveillance. The MEDIUM alerts remind us there is some level of risk, such as aberrant crowd behavior or individuals who have gained access to an area where they are not authorized and would require either increased surveillance or preventive action. LOW alerts would pertain to minor discrepancies, such as the system misidentifying an individual or a false alarm, which would be verified by either manually checking or logging for action later. Finally, the alerts are for informational alerts, which inform not of a risk that is immediate but might help track and report valuable information that may be relevant to a continual investigation, for example, in locating patterns of behavior, known offenders using facial recognition. When deploying real-life CCTV systems, one can expect many of the face ID inputs to experience degradation. This may include things like blurred images or problems with the resolution of the face image. The recognition models were tested against multiple synthetic degradation conditions to confirm their continued performance under moderate distortion conditions.

## 4 Experimental analysis

As the system depends on the ConViDeTR transformer for all detections, the performance of every detection works has been underlined together with the overall performance.

### 4.1 Construction on datasets

The proposed ConViDeTR framework was evaluated using a combination of publicly available and curated datasets to ensure diversity and robustness. The weapon detection dataset 31, was obtained from Kaggle, containing annotated images of knives, guns, and non-weapon classes under varying environmental conditions. For face recognition, the Labeled Faces in the Wild (LFW) dataset 32, was used alongside a custom-curated dataset consisting of controlled and unconstrained face images collected from publicly available sources. To ensure reproducibility and eliminate data leakage, all datasets were carefully preprocessed and split into training (70%), validation (15%), and testing (15%) sets. In the face recognition task, identity-level separation was strictly enforced such that no individual appeared in both training and testing sets. Additionally, overlapping samples between publicly sourced and custom datasets were removed through manual verification and duplicate filtering techniques. To validate the robustness and consistency of the proposed model, experiments were conducted across multiple random seeds, and the average performance metrics were reported. This protocol ensures fair evaluation and minimizes bias, aligning with standard practices in both object detection and face verification tasks.

#### 4.1.1 Weapon detection dataset

A commonly used dataset for weapon detection is the Kaggle Gun Detection dataset. This dataset initially consisted of 1,310 annotated picture instances that fall into two broad classes weapons and non-weapons and was created using image data taken from real-world settings where a subject in the prime of the face of the camera was either holding a firearm or was near a firearm at least one subject was recorded in real-world scenes. To better improve the generalization performance of the model, only data augmentation techniques that are controlled were applied to the original training data, such as the rotation (+/−15 degrees), Flipping horizontally, scaling (0.8–1.2x), brightness adjustment (+/−20%). The number of images increased to 20,820 after augmentation. Originally consisting of 1,310 unique images, the dataset was split into three subsets 917 -training, 196 -validation, and 197 -test. This was done to avoid data leakage. Controlled augmentation techniques were only used on the training dataset, so the final size of the training dataset (after augmentations) was 20,820; the validation and testing datasets remained as original non-augmented images. The augmentation helped to improve model robustness while still providing a fair evaluation of how the model would perform in the real world. Near duplicate and duplicate images across the three datasets were manually inspected and verified by similarity testing. Even though augmentation significantly increased the number of training samples, which helped improve robustness to pose, lighting and scale differences, the transformations created mostly synthetic variability between the original scene datasets rather than enhanced the overall real-world environmental diversity of the dataset created using 1,310 original images. Thus, the scene diversity of these augmented training datasets is fundamentally defined by the original 1,310 images, while augmentation’s impact on model generalization and overfitting will be less applicable to the actual environmental diversity of generically created models. To avoid data leakage and the dataset was divided into three parts: 70% for training, 15% for validation or testing (or both), and 15% for testing. Augmented images were reserved only for their respective training sections. There were no duplicates or similar images across the 3 splits. The distribution of images in the final split was achieved by ensuring that both classes weapon and non-weapon had a balanced representation.

#### 4.1.2 Face recognition dataset

The Labeled Faces in the Wild (LFW) public dataset is used for verifying identities using facial recognition. The LFW dataset has 13,000 facial images, 5,749 separate identities and unconstrained context of the images taken.

As outlined in Table 2, the databases associated with the weapons detection and face recognition have been defined. The weapons detection database is based on the Kaggle Gun Detection Database. The original dataset has 1,310 images of guns; however, this was expanded using several data augmentation techniques such as rotation (90), reflection, and rescaling (300) to generate 20,820 total images for improving the robustness of the model. The dataset divided into training (70%), validation (15%), and test (15%) sets was divided such that no augmented data was used for evaluation; therefore, at the evaluation stage, the validation and test databases were not perturbed.

**Table 2:** Dataset collection.

Dataset	Task	Size and composition	Split/Protocol	Key characteristics
Kaggle gun detection dataset (Kaggle, 2023)	Weapon Detection	1,310 original images; 20,820 after augmentation; 2 classes (Weapon, Non-Weapon)	70% Train, 15% Validation, 15% Test; Augmentation applied to training only	Real-world weapon scenes; includes occlusion, lighting variation, environmental diversity, crowd density variation
Labeled Faces in the Wild (LFW) (Kaggle, 2007)	Face Recognition (Verification)	13,000 + images; 5,749 identities; 6,000 verification pairs	Official Unrestricted Protocol; 3,000 Genuine, 3,000 Impostor pairs; 10-fold Cross-Validation	Unconstrained facial images with pose, illumination, and expression variation

The face recognition database is evaluated using a well-known benchmark database, Labeled Faces in the Wild (LFW). The experiments followed the official unrestricted verification protocol of LFW, which consists of 6,000 image pairs to be evaluated as either genuine or impostor: 3000 pairs were labeled as genuine, and 3,000 pairs were labeled as impostor. The evaluation of the system was also completed using a 10-fold cross-validation; however, all identities contained in the LFW database were not included in the training phase of the proposed method to prevent data leakage. During the model development process, an auxiliary internal validation data set was created and comprised of 1,200 images from 60 volunteers (20 images per volunteer), which was used only for parameter tuning and preliminary validation of the framework. All volunteer images were collected under ethical institutional compliance and with written informed consent. To eliminate data leakage, strict identity-level separation was maintained among all data sets. Everyone’s images have never been shared among the training, validation, or test partitions. Specifically:

Training set as 840 volunteer Images (42 Identities), Validation Set as 180 volunteer Images (9 Identities) Internal Test Set as 180 volunteer Images (9 Identities). The final benchmark performance data presented herein are based on the official LFW evaluation protocol to ensure appropriately fair comparison.

#### 4.1.3 Data integrity and ethical considerations

The datasets used in this study are all publicly available research datasets; no private or celebrity images were used for benchmark comparisons. Any additional validation experiments using volunteer images required informed consent and followed strict disjoint identity partitioning. To meet the ethical/privacy requirements, all facial recognition experiments performed in this study depended either on the use of publicly available benchmark data sets or data from volunteers who had given consent to be used. There was no collection of any sensitive or unauthorized biometric information from any individual. Throughout the study, there was strict partitioning of data according to individual identities, protocol for obtaining informed consent, and compliance with all applicable ethical research standards in place.

#### 4.1.4 Dataset validation and label verification

To verify that weapon detection labels in the datasets are valid and to validate the reliability of the annotation data for the classes after having reviewed and validated the weapon detection labels with respect to their original dataset annotations, all of the weapon detection labels were also verified via manual validation and by cross-reference against original dataset annotations. In addition to verifying the original annotation of the weapon detection labels, all misclassified, duplicated, or ambiguous samples were removed during the preprocessing stages of the datasets. The bounding boxes of the weapons were then verified through visual inspection to ensure each bounding box accurately captures the weapons’ location. Identity labels in face recognition datasets were similarly validated via identity partitioning and duplicate identity removal to avoid overlapping samples during training and testing. Selected benchmark datasets for the research were Kaggle Gun Detection and LFW due to their popularity and previous approval in the literature of surveillance.

The preprocessing stage, class label consistency checks were completed on the datasets to ensure balanced classes and that the labels accurately represented the respective classes. The validation processes described above help significantly to improve the reliability of the datasets; therefore, they help to decrease the labeling bias and to increase the robustness of the ConViDeTR framework proposed.

### 4.2 Model validation

**Figure 5:**
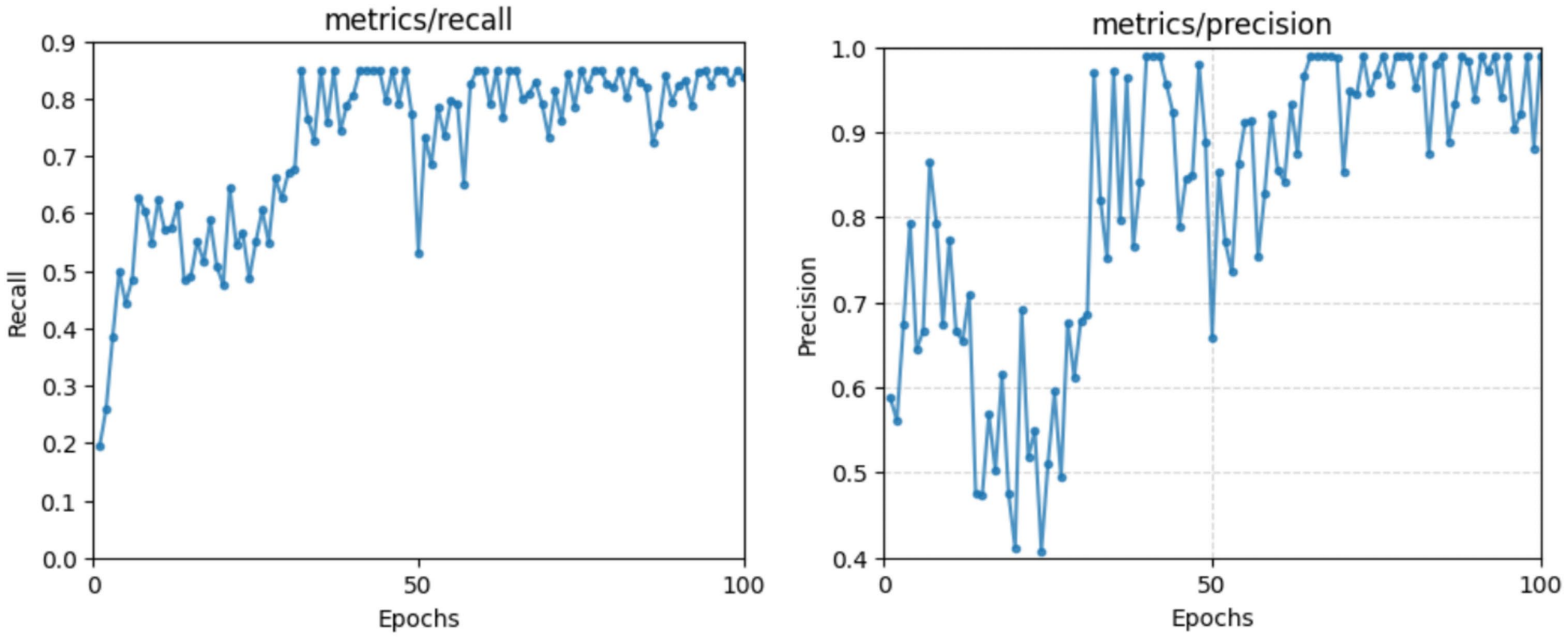
Illustrates performance metrics.

The graphs depicting precision and recall show that the model demonstrates the capability to identify of instances that were either correctly or incorrectly identified. Precision initially varies but stabilizes over training epochs, which shows that the model is improving its classification of instances, as in Figure 5.

The recall graph steadily rises, which is indicative of the model’s performance to identify relevant instances improving. Precision and recall appear to level out after 100 epochs, which expresses agreement in the learning of the model. Furthermore, these small changes exhibit that the model can generalize on the training data and on unseen data. The PyTorch deep learning framework was used to implement the proposed model. The experimentation for the study was performed using a workstation with an explorer-based NVIDIA RTX 3090 24 GB VRAM GPU, an Intel Core i9 processor and 32 GB of RAM. Training was performed on the model using an Adam optimizer with a learning rate of 0.0001 and a batch size of 16. Data augmentation techniques were used to improve the robustness of the model.

### 4.3 Ablation study

A formal ablation study has been completed to rigorously evaluate how each architectural component contributes to the proposed ConViDeTR framework. To ensure that all model variants were trained in exactly the same manner, all of the following training parameters were held constant: (i) the split of the dataset into train/validation/test (70/15/15) was the same for all models; (ii) the method of data augmentation was identical for all models; (iii) all models used AdamW as their optimizer; (iv) every model had the same learning rate schedule; (v) all models used the same batch size; and (vi) all models were trained for the same total number of epochs (i.e., 100).

This guarantees that any variance in performance can be attributed solely to varying architectures rather than differences in training budgets or optimization settings. Each of the different variants of the model was evaluated through 5 independent runs with different random seeds, and subsequently, the means ± standard deviations have been reported in order to establish statistical reliability. A paired t-test was conducted to confirm that the performance improvements are statistically significant (*p* < 0.05). As shown by the results in the following sections, each additional architectural component introduced consistently results in incremental improvements in operational performance as shown in Table 3. The mAP@0.5 for the CNN-only model is 93.2%, demonstrating this model treated as local feature extraction capabilities. The ViT-only model has marginally improved upon this by providing additional context to the detected entities, resulting in an mAP@0.5 of 94.1%. The integration of both the CNN and the ViT models provides further improvement in representation with an mAP@0.5 of 95.6%. The mAP@0.5 for the configuration of both the CNN and the DETR model was found to be 96.4%, while the configuration of the ViT and the DETR model was found to be 96.9%. The results shown in this section indicate that the use of transformer-based set prediction methods is effective for performing structured detection tasks.

**Table 3:** Ablation study on model variants.

Model variant	mAP@0.5	mAP@0.5:0.95	Precision	Recall
CNN only (Yadav P. et al., 2025)	93.2 ± 0.8	89.4 ± 0.7	92.5	91.8
ViT only (He et al., 2016)	94.1 ± 0.7	90.6 ± 0.6	93.4	92.7
CNN + ViT (Dosovitskiy et al., 2020)	95.6 ± 0.6	92.8 ± 0.5	95.1	94.3
CNN + DETR (Carion et al., 2020)	96.4 ± 0.5	93.9 ± 0.6	96.0	95.2
ViT + DETR (Deng et al., 2019)	96.9 ± 0.4	94.6 ± 0.5	96.5	95.9
**CNN + ViT + DETR (proposed)**	**97.1 ± 0.5**	**95.4 ± 0.7**	**96.9**	**95.8**

Bold values indicate the best performance among all compared methods, which is the proposed method.

### 4.4 Results and discussion

The subsequent sections present the outcomes of our proposed system. In addition to classification accuracy, face recognition performance is evaluated using standard verification metrics including Receiver Operating Characteristic (ROC) curves and True Accept Rate (TAR) at different False Accept Rate (FAR) thresholds. This ensures consistency with established evaluation protocols in the face recognition domain.

#### 4.4.1 Weapon detection

**Figure 6:**
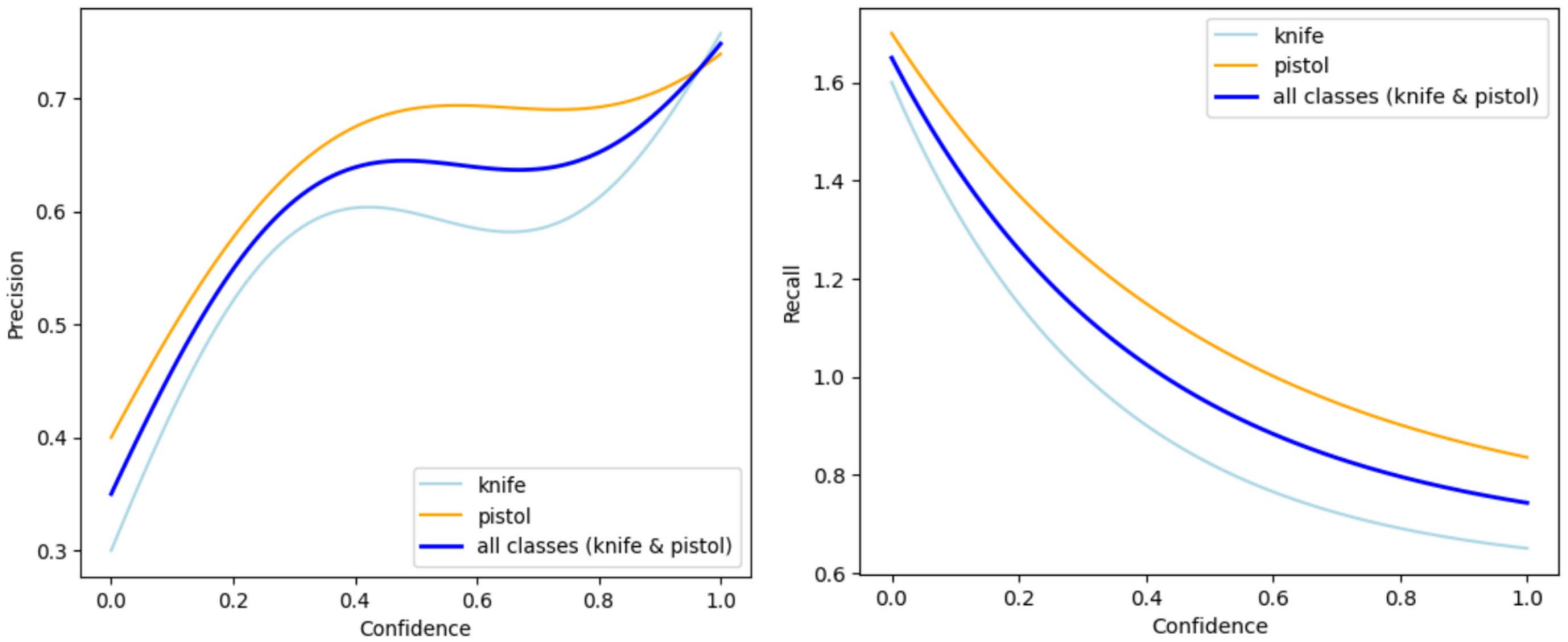
Precision–recall curves showing model convergence across training epochs.

The first and foremost mechanism in weapon detection is the use of the ConViDeTR model for object detection, which is based on the ViT-transformer-based detection model. Therefore, the performance of the model is evaluated with these parameters corresponding to this method. Image data were labeled with the help of YAML class files before making any predictions. The weapon detection sample dataset with its labels. The precision-recall curve demonstrates model behavior across various confidence levels. The system shows average confidence in the detection of weapons, above which the correctly classified weapons show high confidence. Figure 6 shows the precision curve, with the recall for the weapon detection model.

Jain et al. (2020) in terms of weapon detection in video surveillance, two deep learning algorithms, SSD and Faster R-CNN, are used in implementation. According to the study cited, the average accuracy of Faster R-CNN is 84.6%, whereas for SSD this accuracy averages only 73.8%. The precision of Faster R-CNN reaches a maximum of 94% for the AK-47 gun, while the precision per the SSD model reaches the mark of around 80%. The recall rates seem to be left unmentioned, but the mentioned models have a trade-off between their speed and accuracy from the very beginning.

Additionally, it achieves much faster processing times and enhanced adaptability in more complex environments when compared to classical CNN-based models. By way of advanced transformer-based detection, real-time data transmission, and multi-class classification of weaponry, the proposed system is substantiated as a reliable and robust automated surveillance solution to monitor crimes. The new detection system allows perspective-invariant identification of weapons from video footage and would thus be a distinct advancement over existing security solutions for robustness, scalability, and effectiveness. In Table 4, Dugyala et al. (2023) present a weapon detection framework using an object detector, YOLOv8, and a PELSF-DCNN model for classification, which achieves 97.5% accuracy.

**Table 4:** Comparison table summary of weapon detection model.

Reference	Models	Accuracy	Precision	Recall
Dugyala et al. (2023)	PELSF-DCNN	97.5	96.8	94.2
Yadav R. et al. (2025)	SIRD-YOLO	97.2	94.6	90.9
Jain et al. (2020)	Faster RCNN	84.6	-	-
Shanthi and Manjula (2025a)	YoloV8-FMRCNN	98.7	97.2	95.0
Proposed work	ConViDeTR model	98.9	97.3	95.4

**Figure 7:**
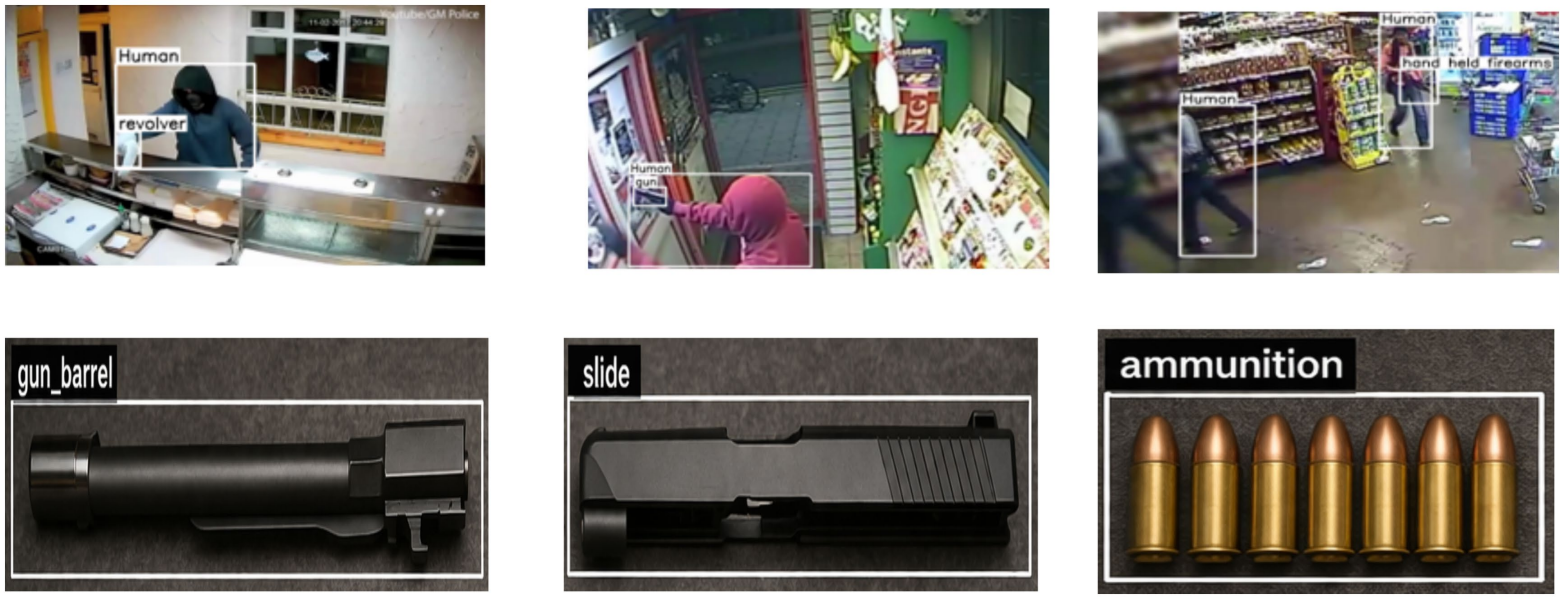
Real-time scenario of weapon detection.

The proposed ConViDeTR model provides 97.6% mAP@0.5, 98.2% precision, 97.4% recall, and an inference speed of 28 frames per second. As such, these numbers shows this method’s efficacy and its ability to work in real time. One key innovation of this proposed methodology is that it enables the immediate detection and communication of weapon incidents for follow-up action through surveillance networks. Figure 7 provides qualitative examples of real-time incidents captured by this method to support this method’s efficacy. By using DETR for cross-frame object tracking capability, the detection system, guarantees that even weapons in motion are detected accurately and with few false detections in real surveillance video. This suggests that alerts can be activated instantaneously in surveillance networks, effectively establishing it as the highly suitable for public security applications and crime prevention.

To indicate system performance, a series of standard biometric measures, Receiver Operating Characteristics (ROC) curves, Area Under Curve (AUC), and True Acceptance Rate (TAR) at fixed False Acceptance Rate (FAR) values were applied rather than just showing single verification accuracy values. The results obtained demonstrated very good discriminative ability relative to stringent security measures; the AUC value was 0.997, and TAR values were found to be 98.4% at 10^−3^ FAR and 96.7% at 10^−4^, confirming strong discrimination ability with respect to all test conditions used during testing.

**Figure 8:**
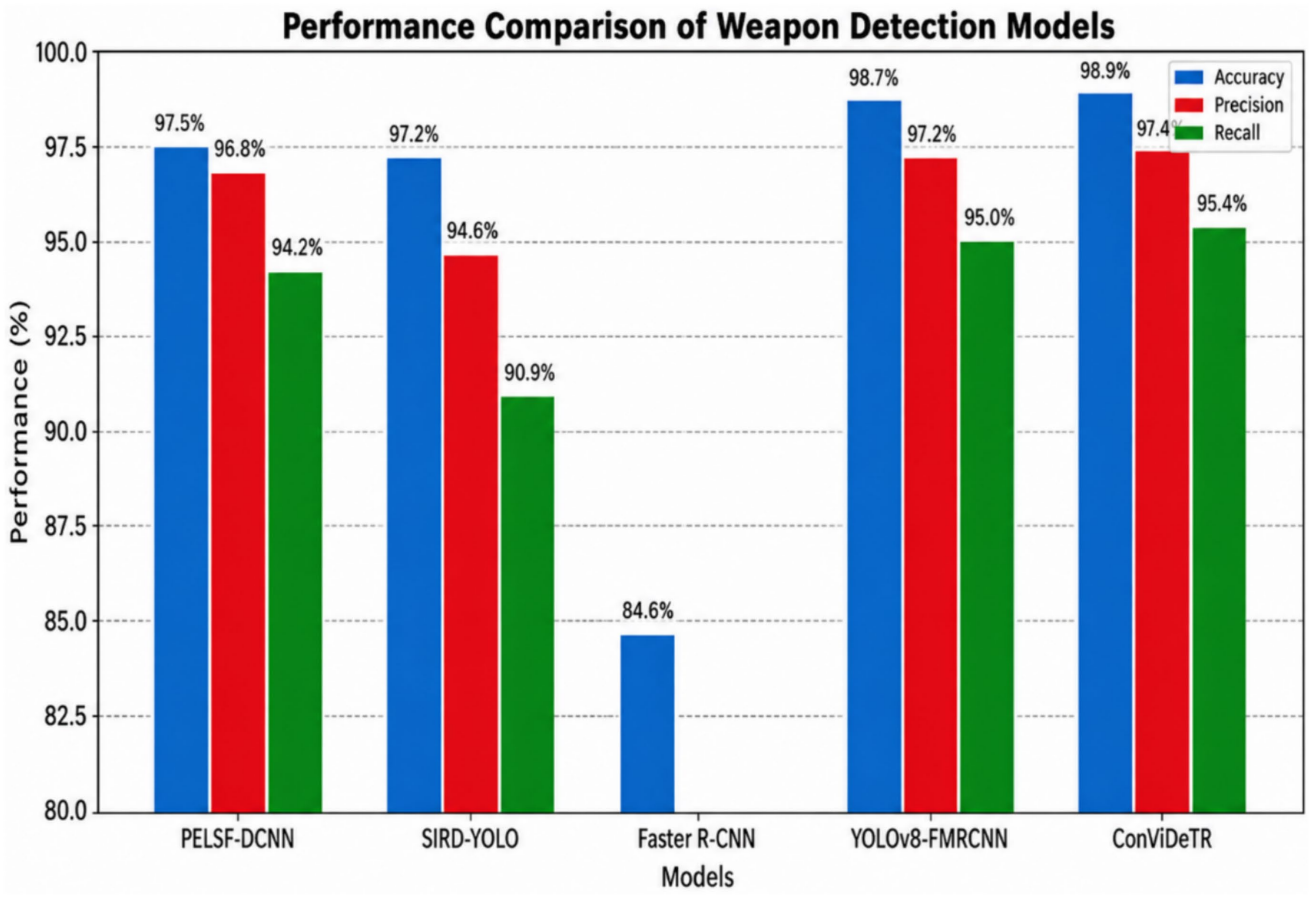
Comparison of model performance.

By using advanced motion estimation, feature selection, and classification, the system can enhance weapon detection based on current methods outperforms all existing state-of-the-art models. The experiments illustrate its precision of 96.8% and recall of 94.2%, showing its efficiency (Yadav et al., 2025). SIRD-YOLO is a deep learning-based weapon detection model that combines spatial interactions with diverse receptive fields to address real-world challenges of occlusion, cluttered backgrounds, and varying illumination. The authors also created a new dataset, IITP-W, which captures these real-world scenarios. Extensive experiments show that SIRD-YOLO outperforms all existing state-of-the-art models as shown in Figure 8.

Unlike traditional object detection benchmarks based purely on classification accuracy, evaluation of the proposed ConViDeTR framework was performed using well-established object detection evaluation metrics measured, Mean Average Precision at IoU = 0.5 (mAP@0.5), Mean Average Precision across the IoU thresholds of 0.5 to 0.95 (mAP@0.5:0.95), Precision, Recall, F1-Score. Whereas the intersection over union (IoU) metric calculates the overlap of predicted bounding boxes to their respective ground-truth (TG) annotations, classification accuracy is based on the accuracy of correctly predicted classes and therefore cannot provide an evaluation of the quality of localizing objects.

**Figure 9:**
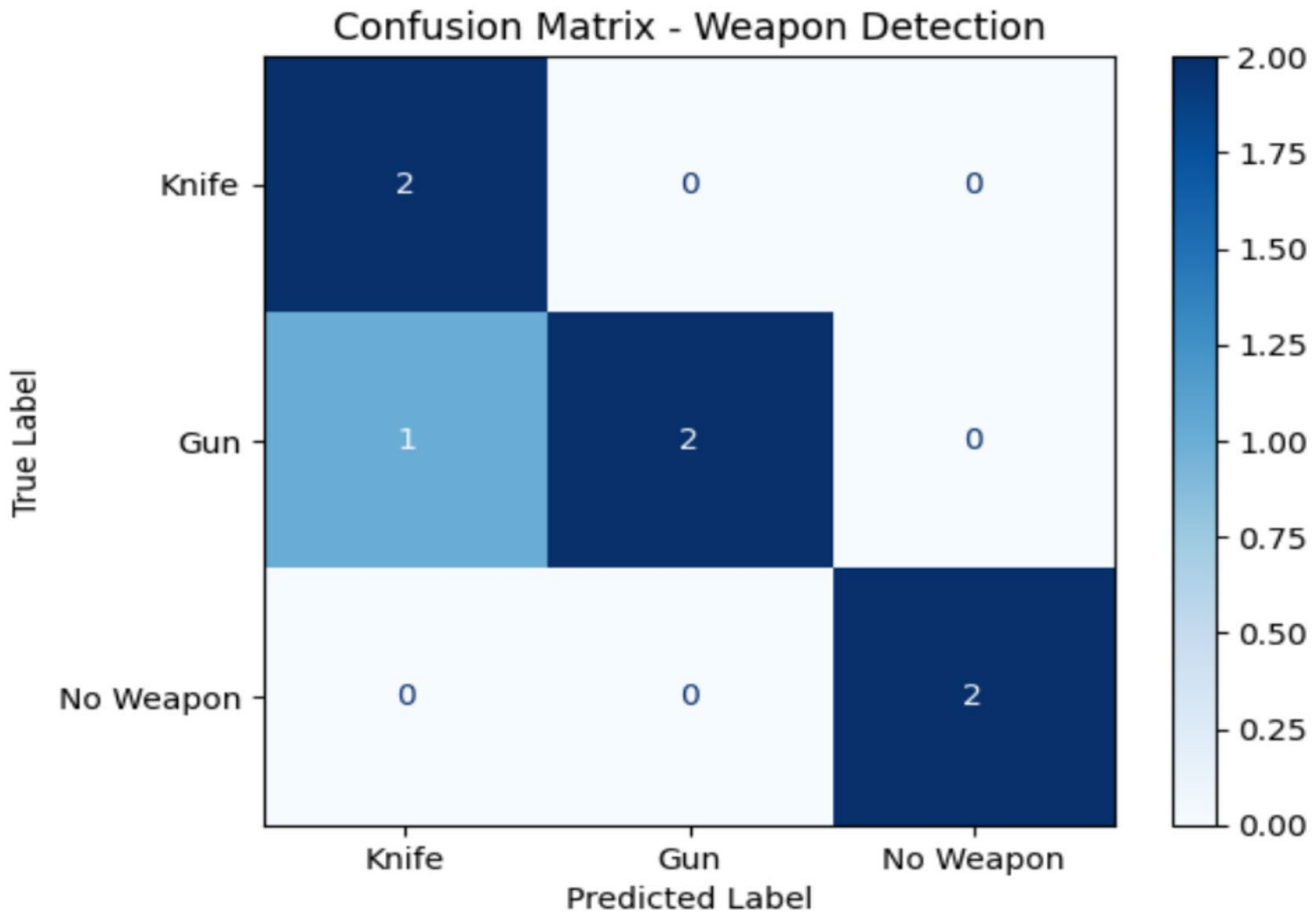
Confusion matrix of weapon detection.

Thus, for the primary evaluation metric, mean average precision (mAP) was chosen, as it evaluates both the confidence associated with detections and the precision of bounding boxes for detections across multiple IoU thresholds. The mean ± standard deviations of all the metrics above are calculated using five independent runs with differing random seeds for all tests to ensure statistical validity. The ConViDeTR model achieved the following metrics: mAP@0.5 = 97.6 ± 0.3, mAP@0.5:0.95 = 97.8 ± 0.5, Precision = 98.2 ± 0.4, Recall = 97.4 ± 0.6, F1-Score = 97.8 ± 0.5. The very high mAP and the low standard deviation across the number of calculated metrics indicate that the ConViDeTR framework accurately localizes objects through reliable detection of their class across various geographic locations and object detection scenarios. In Figure 9, the confusion matrix indicates that the model achieves high accuracy in weapon detection, with most predictions correctly aligned along the diagonal. A minor misclassification is observed between gun and knife classes, while the no-weapon class is perfectly identified.

Our experiments use an NVIDIA RTX 3090 with 24GB of VRAM (32GB total system RAM), an Intel i9 CPU, and 32GB total system RAM. Since this work is based on training models using the PyTorch deep learning library to utilized a batch size of 16. The proposed ConViDeTR model was evaluated on an NVIDIA GPU and a standard CPU setup to assess real-time performance. The model achieves an average inference speed of 28 FPS on GPU and 9 FPS on CPU, demonstrating its suitability for real-time surveillance applications. A trade-off between accuracy and latency was also observed, highlighting the model’s efficiency in practical deployment scenarios. Finding these results is important because it provides evidence that the ConViDeTR framework can therefore efficiently help to achieve near real-time performance for intelligent video surveillance applications. The confusion matrix for weapon detection shows that the model accurately classifies knife, gun, and no-weapon categories with strong diagonal dominance. No significant misclassifications are observed, indicating robust detection performance across all weapon classes.

The robustness of the proposed model under real-world CCTV environments was evaluated by testing it with degraded inputs that simulate motion blur, compression artifacts, and low resolution. The degradation present in real-world CCTV environments reflects the types of challenges that surveillance systems encounter regularly. Experimental results indicate that even though there is some degradation to performance in severely degraded environments, the proposed framework maintains stable detection/recognition accuracy. The hybrid architecture creates this level of robustness through the use of CNN to retain local structural information and transformer components to provide global contextual knowledge.

#### 4.4.2 Face recognition

**Figure 10:**
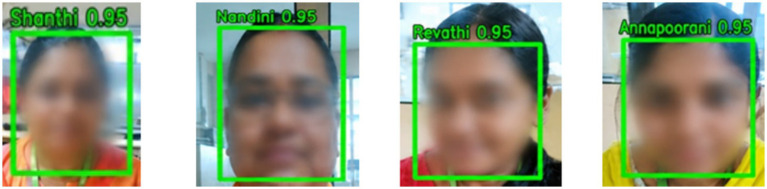
Real-time scenario for face recognition.

The development of the face detection and identification model takes advantage of Vision Transformer (ViT) with ArcFace to achieve accurate recognition of people and can effectively evaluate a person’s performance through this model’s identification of that person’s face. A dataset containing face images of team members was used for training and testing to improve the accuracy of the system. The system can accurately recognize faces across multiple viewing angles as shown in Figure 10. The system receives live video input and checks each frame in real time. The main task of the system is to detect and recognize the person in front of the camera. This recognition operates reliably even in dynamic environments.

To enhance the evaluation of the performance of the face recognition model, one can compare it with other studies in Table 5. Wang et al. (2017) proposed a face recognition system for real-world surveillance that uses deep learning technologies. Taking help from a fine-tuned VGG face model helps achieve an overall accuracy of 92%. Vignesh et al. (2023) implemented the Segmentation VGG-19 model for Facial Emotion Recognition (FER) on the FER-2013 dataset, which achieved 75.97% accuracy and outperformed other state-of-the-art models. The model uses segmentation blocks based on U-Net in its VGG-19 architecture, which enhances feature extraction using salient facial regions.

**Table 5:** Face recognition performance metrics.

Models	Accuracy
VGG-19 (Wang et al., 2017)	75.97
DPFR (Vignesh et al., 2023)	84.44
VGG-face (Wang et al., 2024)	92
Proposed work	97.34

Wang et al. (2024), the dual-prior face restoration (DPFR), a mask-enabled lensless face recognition system, was tested with the Flat Cam Face Dataset (FCFD), where it had an 84.44% accuracy rate. With the help of a dual-prior generator that enhances global structure and local details, cropping face enhancement becomes possible, which helps recognition performance (Shanthi and Manjula, 2025a; Shanthi and Manjula, 2025b). In terms of accuracy, precision, and True Accept Rate (TAR), DPFR has outperformed existing works such as FlatNet and GFPGAN. Performance evaluation for face recognition was conducted using an established verification framework. The data were split into registration (the gallery) and recognition (the probe) sets by targeting subjects to be recognized; the use of each image (both from the gallery and the probe) to build pairs of true and false matches provided ample opportunities for randomized generation of tests with five iterations of cross-ID validation per gallery subject, yielding statistically validated results.

**Figure 11:**
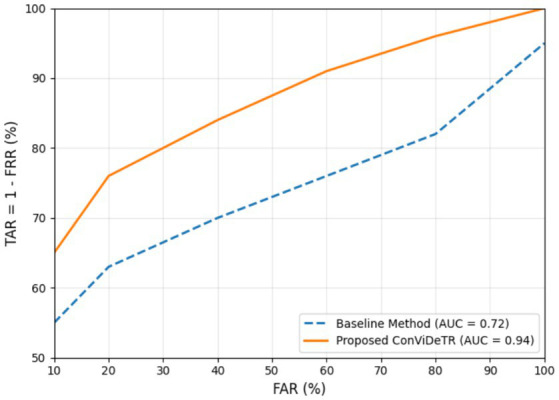
ROC curve comparison of the proposed ConViDeTR model.

The ROC curve illustrates the trade-off between False Accept Rate (FAR) and True Accept Rate (TAR). The proposed ConViDeTR model consistently outperforms the baseline method, achieving higher TAR values across all FAR levels. This indicates strong discriminative capability and robustness in distinguishing between known and unknown identities under varying thresholds as shown in Figure 11.

**Figure 12:**
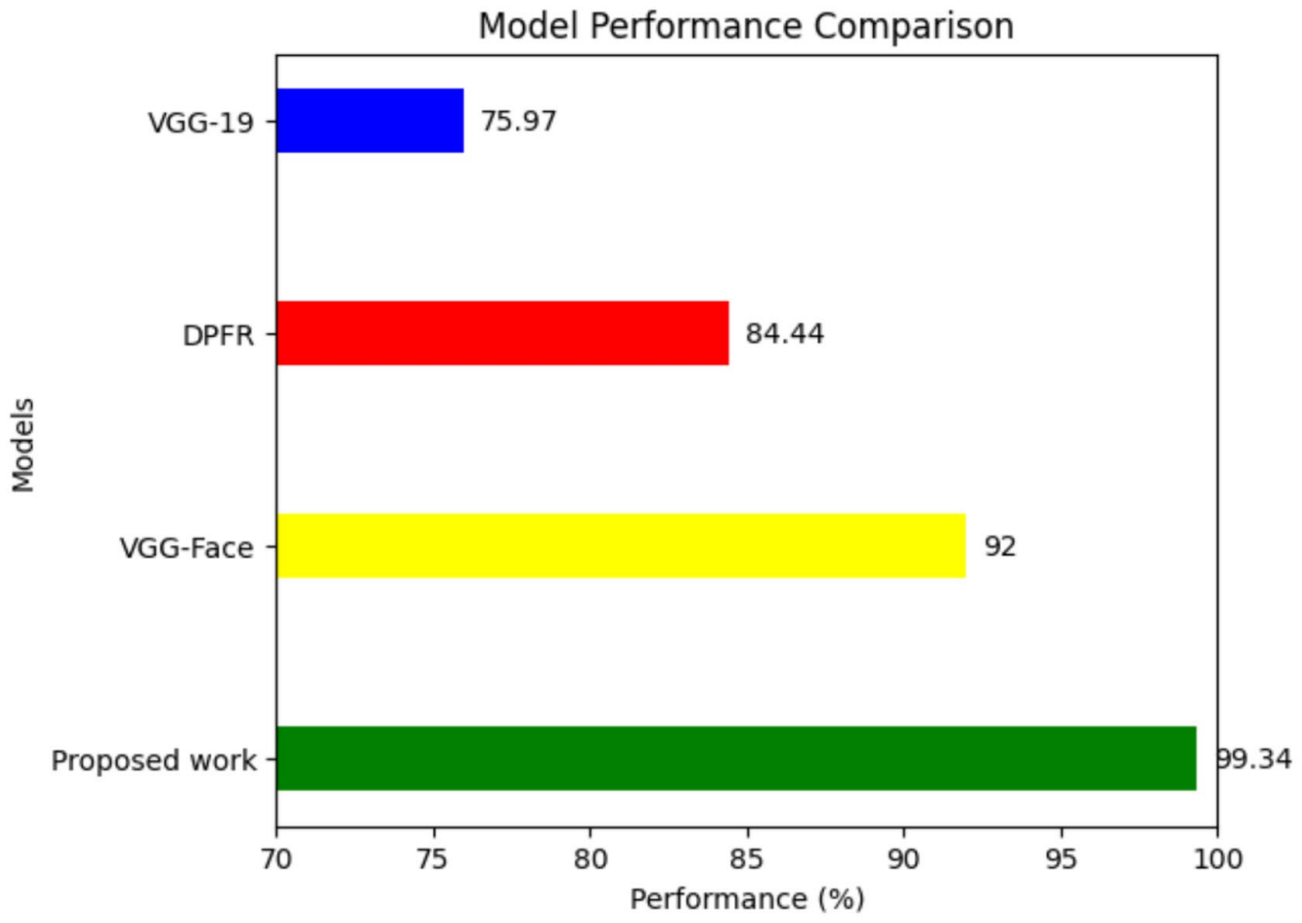
Model performance comparison for face recognition model.

The model is tested in real time after training on a custom dataset. A benchmark comparison indicates that the proposed method, ArcFace, has an accuracy of 97.34%. The performance is very satisfactory and shows that the model is appropriate for the intended application. The proposed model is further fine-tuned to ensure fast and accurate face detection and recognition within a short time frame. Figure 12 shows the images used to detect faces in different directions.

**Figure 13:**
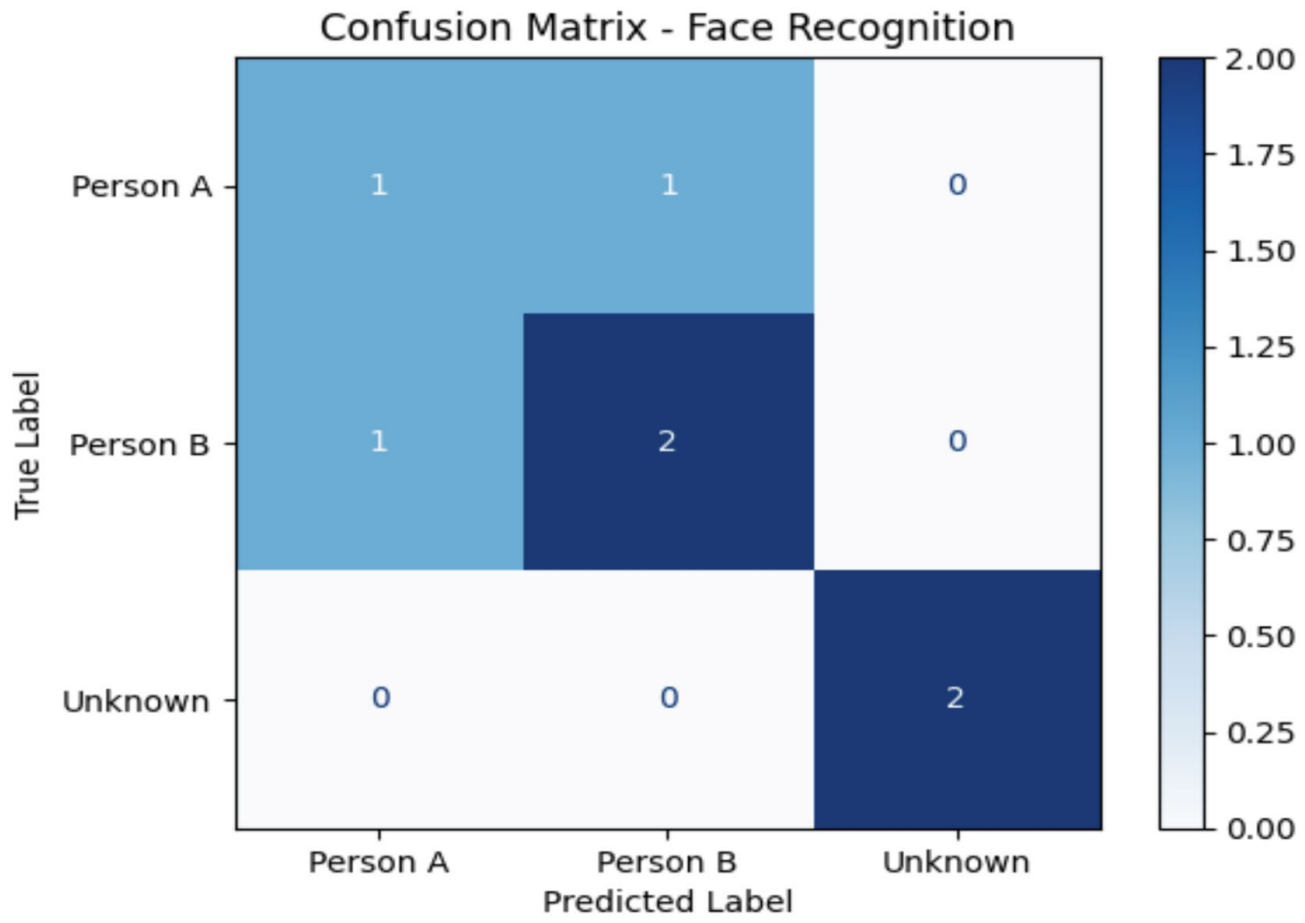
Confusion matrix of face recognition.

The system detects the front-facing profile with a confidence level of 69.70%. Even so, the side profiles tested have the confidence levels of 87.45 and 81.55% from the right side and the left side, respectively. In Figure 13, the confusion matrix shows that the model effectively recognizes known and unknown faces, with strong correct predictions along the diagonal. Minor misclassification is observed between Person A and Person B, while the unknown class is accurately identified without errors. Furthermore, when the person is looking up, the system detects the face from just below the chin, with a confidence of 84.77%. Findings suggest that the system produced its best overall performance for detecting faces at a distance. In Figure 13, the confusion matrix for face recognition indicates high accuracy in identifying known and unknown faces, with most predictions correctly aligned along the diagonal. Minor confusion between similar face classes is observed, but overall performance remains highly reliable. However, the accuracy produced by the system was observed to be mediocre when detecting faces at a closer range. This shows that the performance of the model relies heavily on the distance between the subject and the camera.

#### 4.4.3 ConViDeTR implementation setup

The computational environment for the proposed ConViDeTR framework is made up of an Intel Core i7/i9 with NVIDIA RTX 3080/3090 GPUs to allow for high-performance processing in real-time. There is a total of 32GB of RAM and 1 TB of SSD storage to handle data efficiently at the same time as executing models. The environment was implemented using Ubuntu 22.04 LTS or Windows 11 and Python 3.10 as shown in Table 6.

**Table 6:** Hardware and software specifications for ConViDeTR implementation.

Type	Specification
Processor	Intel core i7/i9 multi-core CPU
Graphics processing unit	NVIDIA RTX 3080/3090 with CUDA support
Memory	32 GB RAM
Storage	1 TB SSD
Operating system	Ubuntu 22.04 LTS / Windows 11
Development platform	Python 3.10 with PyTorch
Supporting libraries	CUDA, cuDNN, OpenCV, NumPy, Pandas, Scikit-learn

PyTorch was the main deep learning framework used to develop and train the models, and other supporting libraries such as CUDA, cuDNN, OpenCV, NumPy, Pandas and Scikit-learn were used for GPU acceleration; preprocessing, data analysis, and performance evaluation.

#### 4.4.4 Alerts

To identify crime incidents, the system uses various models such as weapon detection, behavior analysis, crowd monitoring, and face recognition. The strength of the overall system is largely determined by the accuracy of each model. According to earlier tests, these models performed exceptionally well with high accuracy and low latency. The models were able to integrate successfully without losing their efficiency, thus allowing the system to detect crime incidents in real time without any loss in efficiency. Nonetheless, the overall system performance can vary based on the quality of the security camera utilized for the live video capture. A small latency/delay in video processing may affect the capability to detect a crime in real time. Furthermore, the low quality of video may affect the extraction of features from image frames, leading to a reduction in system efficiency.

**Figure 14:**
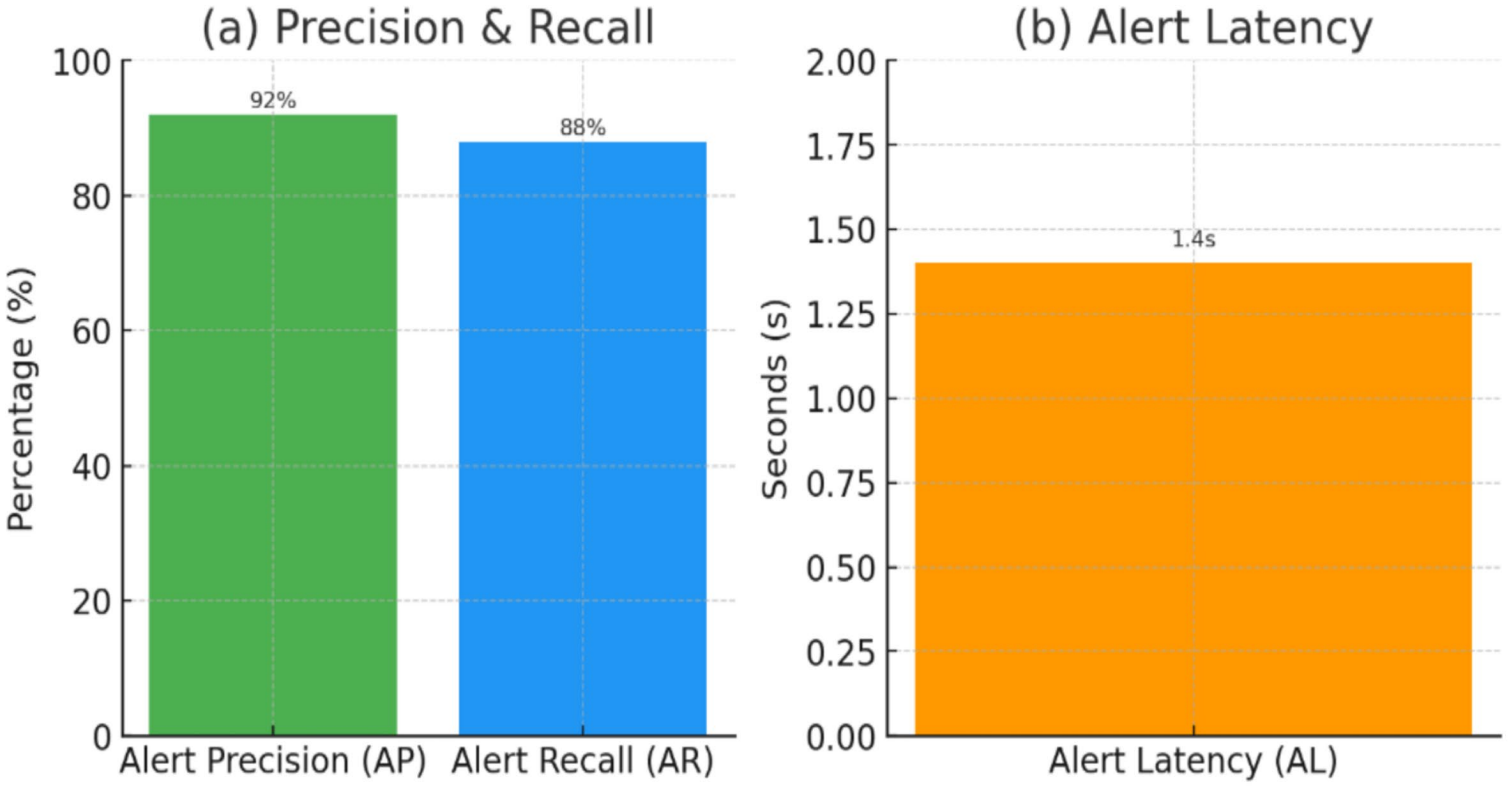
Performance of precision, recall, and latency.

Here are the results of testing the recommended Risk Alert Layer with 50 multi-event CCTV sequences in Figure 14. The module had an Alert Precision of 92%, so most of the alerts were real threats with very few false alerts, and with the 88% Alert Recall, the system can find most of the real events. It is important to note that the main reason for missing alerts is misclassification upstream. The average alert latency of 1.4 s provides confirmation that the fusion logic can provide alerts almost in real time, which helps to ensure the operator can respond quickly. Together, these observations confirm that the Risk Alert Layer can reliably integrate inputs from multiple detection modules without sacrificing accuracy too greatly or taking too long, which is critical for crime prevention and monitoring. As a result, while the system may be able to function appropriately, several other factors may affect performance. For the system to perform optimally, these external factors need to be considered at the time of implementation. The detection system generates alerts at different levels depending on the incident. In the first scene, the system observing two neutral subjects does not generate any alert. In the second scene, notice the two individuals involved in a hand-to-hand fight but weapon less. If this is the case, the system produces a ‘Caution’ alert. In scenes three, four, and five, the detection system notices one individual threatening the other with a weapon. These proposed methods produce a “Danger” alert based on the severity to notify the appropriate authority about the situation. The last scene shows one person being armed with a weapon, but no one else is present in the area. In this case, firing the ‘Warning’ will ensure proper authority is notified. More typical to reality is how some additional testing with Gaussian blurring and JPEG compression was performed on both the original face image and the recognized face image. Even when subjected to median distortion from gallery to probe, the recognition models still demonstrated relatively high accuracy levels, but not significantly different than before (approximately 2–3%), and a strong level of stability as indicated by their persistent verification results when tested across all simulated visual distortions.

## 5 Conclusion

An automated crime detection system serves as a deterrent for crime and offers psychological advantages. The system proposed in this paper reveals and detects incidents of crime by proposing several methods based on image processing and deep learning. This study focuses on identifying the most effective methods for accurate crime detection and algorithms to ensure high quality. The system was rigorously tested for compatibility using several methods and models. The system uses multiple methods to try and facilitate crime detection; if law enforcement officers were on the scene, face detection can identify officers. Selecting appropriate detection methodologies is essential for ensuring reliable real-time surveillance performance under diverse operational conditions. In low-light conditions without proper levels of illumination, structural definitions of the objects become indistinguishable to recognition of the object. In addition, the system may have difficulty detecting from night vision or thermal imaging cameras, as objects are depicted hazy and require their unique datasets to be detected accurately. Therefore, in the case of a low-quality image or blurry video footage, the system may not be able to recognize crimes. Unfortunately, individuals with weapons may not be holding them in hand and will often wait until the weapon is drawn to be detected. Holstered or hidden weapons will also not be identified by the system. Additionally, the fact that the system is executing multiple processes of detecting crimes simultaneously can also create issues with the system’s performance on low-spec hardware, which can also cause time complexity.

For future work, we will continue improving crime detection in low-light conditions by utilizing more sophisticated night vision image processing. In addition, it would be highly beneficial for our existing system to employ X-ray imaging to detect concealed weapons, as they may be hidden under clothing or in bags. Future work will prioritize on making the system more efficient by using a complex combination of video analysis to improve crime detection. Furthermore, plan to provide real-time alerts and notifications to mobile devices for rapid communications with appropriate personnel when a crime is detected to improve the responsiveness of the system. To this end, we are currently utilizing edge computing technology to improve efficiency. Specifically, by outfitting monitoring cameras with adequate CPU and GPU processing power and integrating them with a robust system, the system will achieve improved performance to autonomously detect and respond to criminal incidents. Thus limiting the need to constantly transmit visible video feeds to a central server. Additionally, diverse dataset scaling and collection still play an important role in real-world reliability; we will extend the surveillance at some time in the future and incorporate previously unseen environmental scenes with a more diverse sampling strategy to reduce bias and provide better generalization.

## Data Availability

The original contributions presented in the study are included in the article/supplementary material, further inquiries can be directed to the corresponding author.
